# Comprehensive chemical analysis of polyphenols in the ethyl acetate extract from the roots of *Ephedra sinica* Stapf and evaluation of its therapeutic effects on SU5416/hypoxia-induced pulmonary arterial hypertension rats

**DOI:** 10.1186/s40643-025-00963-9

**Published:** 2025-10-29

**Authors:** Mengying Lv, Jinhao Shuai, Yang Wang, Qianwen Lu, Xinlong He, Junjie Zhen, Ling Ling, Jun Yao, Fengguo Xu

**Affiliations:** 1 School of Traditional Chinese Medicine, Faculty of Medicine, Yangzhou University, Yangzhou, 225001 China; 2 The Key Laboratory of Syndrome Differentiation and Treatment of Gastric Cancer of the State Administration of Traditional Chinese Medicine, Yangzhou, 225001 China; 3 Guangling College, Yangzhou University, Yangzhou, 225001 China; 4 School of Pharmacy, Xinjiang Medical University, Urumqi, 830017 China; 5 Key Laboratory of Active Components and Drug Release Technology of Natural Medicines in Xinjiang, Xinjiang Medical University, Urumqi, 830017 China; 6 Key Laboratory of Drug Quality Control and Pharmacovigilance (Ministry of Education), China Pharmaceutical University, Nanjing, 210009 China

**Keywords:** Polyphenols, Root of *Ephedra sinica* Stapf, SU5416, Pulmonary arterial hypertension, Gut microbiota

## Abstract

Pulmonary hypertension (PH) is a deadly disease with limited treatment options and poor long-term survival, necessitating the discovery of novel therapeutics. Our previous study has revealed that dimeric proanthocyanidins (PACs) mainly existed in the ethyl acetate extract from the roots of *Ephedra sinica* Stapf (ERE), however, its therapeutic effects on SU5416/hypoxia-induced pulmonary hypertension (PH) rats remain elusive. In this study, column chromatography combined with UPLC-LTQ-Orbitrap-HRMS analysis was performed to comprehensively characterize polyphenols in ERE. The therapeutic effects of ERE were investigated using the SU5416/hypoxia rat model, in which the rats were injected with SU5416 (20 mg/kg), followed by a three-week hypoxia exposure (10% O_2_). Hemodynamic indicators determined by right heart catheterization, pulmonary arterial morphological changes assessed by histopathological analysis, cardiac function and pulmonary hemodynamics using echocardiography, as well as oxidative stress markers measured by corresponding kits were used to test the therapeutic effects of ERE. Moreover, 16S rRNA sequencing combined with untargeted metabolomics was employed to capture changes in gut microbiota and serum metabolites after ERE treatment. Comprehensive chemical analysis of polyphenols in ERE revealed various levels of proanthocyanidin monomers, dimers and trimers, especially A-type dimers. In vivo experiments showed that ERE decreased pulmonary arterial pressure, right ventricular hypertrophy, right ventricular free wall (RVFW) thickness and oxidative stress levels, increased pulmonary acceleration time (PAT) and alleviated pulmonary vascular remodeling in rats exposed to SU5416/hypoxia treatment. Meanwhile, ERE improved gut microbial dysbiosis and the disturbed glycerophospholipid metabolism. Collectively, this study presents the first report on the efficacy of A-type PACs from *Ephedra sinica* for the treatment of PH through regulating gut microbiota and host metabolism.

**Graphical abstract:**

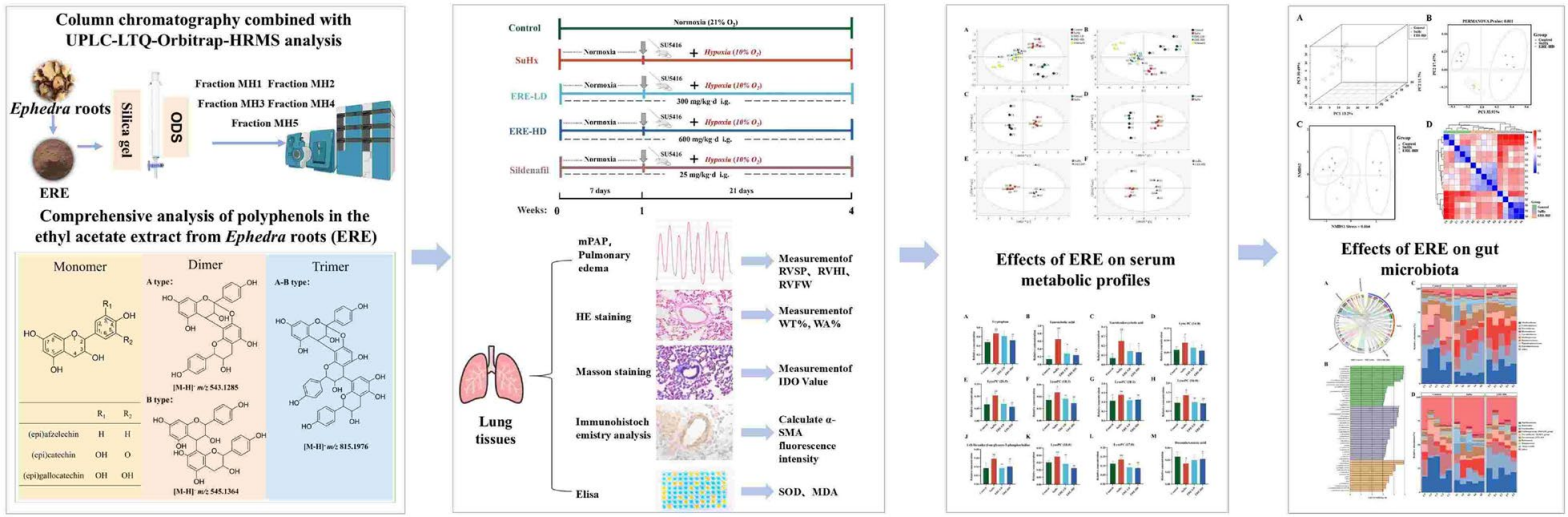

**Supplementary Information:**

The online version contains supplementary material available at 10.1186/s40643-025-00963-9.

## Introduction

Pulmonary hypertension is a progressive and life-threatening disorder characterized by elevated pulmonary arterial pressure and pulmonary vascular remodeling, which resulted in right heart failure and premature death (Fiorentù et al. 2025). PH can be caused by multiple clinical settings such as chronic obstructive pulmonary disease (COPD), idiopathic pulmonary fibrosis and thalassemia (Hadi et al. 2023; Nathan et al. 2019; Wood 2022). Despite significant advances in elucidating the molecular mechanisms underlying PH pathogenesis, such as endothelial dysfunction, smooth muscle cell proliferation, inflammation, and oxidative stress—the disease remains incurable, and patients mainly rely on vasodilators and anti-proliferative mediators to relieve clinical symptoms such as breath shortness, fatigue and syncope (Poch et al. 2021). Therefore, it’s urgent to develop safe, effective and low-cost drugs for the treatment of PH.

Increasing evidence highlights the role of oxidative stress and gut-lung axis disruption in the development and progression of PAH, suggesting that therapeutic strategies targeting redox imbalance and gut microbiota-metabolite interactions may hold promise (Mocumbi et al. 2024; Suswał et al. 2023). Natural polyphenolic compounds, especially PACs, are well-recognized for their potent antioxidant, anti-inflammatory, and endothelial-protective properties (Joo Sul et al. 2024). Among these, flavan-3-ol-based oligomers derived from medicinal plants have attracted increasing attention as potential therapeutic agents for cardiovascular diseases (Feldman et al. 2025; Wang et al. 2024a). *Ephedra sinica* Stapf, a traditional Chinese medicinal herb, is widely known for its bioactive constituents including alkaloids and flavonoids. Our previous comparative chemical analysis of the stems and roots derived from the *Ephedra sinica* Stapf indicated that higher levels of macrocyclic spermine alkaloids and dimeric PACs existed in *Ephedra* roots (Lv et al. 2015). PACs have been demonstrated to possess pronounced blood pressure-lowering effects in clinical investigations, and the mechanism was associated with improving vascular function, preventing oxidative stress and inflammation, and modulating lipid metabolism (Fan et al. 2022). Grape seed PACs was proved to attenuate hypoxic pulmonary hypertension through suppressing oxidative stress and the proliferation of pulmonary arterial smooth muscle cells (Jin et al. 2016). In cigarette smoke-exposed pulmonary arterial hypertension rats, grape seed PACs was found to inhibit inflammation by modulating PPAR-γ/COX-2 pathway (Liu et al. 2020). It is noteworthy that grape seed mainly contained B-type PACs, in which the flavan-3-ols are linked through one interflavan bond (Jin et al. 2016). In the nature, only a few kinds of food such as cranberry possess A-type PACs (one interflavan bond and one ether bond between composing units) and its therapeutic effects on PH has been rarely reported (Sintara et al. 2020). We previously find that the ethyl acetate extract from the roots of *Ephedra sinica* Stapf (ERE) were rich in A-type PACs, however, its therapeutic effects on SU5416/hypoxia-induced PH rats remains unknown, especially from the aspect of metabolomics and gut microbiota.

With the emerging role of multiple omics and bioinformatic analysis applied to exploring the molecular mechanism of various diseases, significant advances have been made to characterize “omics profiles” in PH (Harbaum et al. 2020). According to a recent plasma metabolomics study of 117 participants with PAH, polyamine, histidine, and sphingomyelin metabolic pathways were identified as promising candidates for indicating disease progression and prognosis (Pi et al. 2023). For the past decade, the gut microbiota has been implicated as a leading factor contributing to many diseases. The altered gut microbiota profile was observed in patients with PH, and the “gut-lung axis” offers a novel angle for PH pathogenesis (Kim et al. 2020).

In this study, comprehensive chemical analysis of polyphenols in ERE was performed for the first time using integrated column chromatography and UPLC-LTQ Orbitrap MS. Then the preventative effects of ERE on PH was investigated using the SU5416/hypoxia model, in which the rats were treated with SU5416 and a subsequent three-week hypoxia exposure. Right heart catheterization, histopathological and immunohistochemical analysis as well as echocardiography was performed to evaluate its effects on hemodynamics and pulmonary arterial morphological changes. Furthermore, the UPLC-LTQ Orbitrap MS based metabolomics and 16S rRNA sequencing techniques were applied to capture subtle metabolic changes and gut microbial alterations of PH rats after ERE treatment. Our research will not only promote better utilization of *Ephedra* species, but also provide insights into the development of novel anti-PH drugs.

## Material and methods

### Chemicals and reagents

PEG-300 and Tween 80 were obtained from J&K Scientific Technology Co. Ltd. (Shanghai, China). Sildenafil citrate was purchased from Dongyangguang Pharmaceutical Co. Ltd. (Guangdong, China). SU5416 (4LSOH-SR) was purchased from Tixiai Co. Ltd. (Shanghai, China). The sodium carboxy-methylcellulose (CMC-Na) was supplied by Aladdin (Shanghai, China). HPLC-grade methanol, acetonitrile and formic acid was obtained from Fisher Chemical (USA). The analytical-grade methanol, ethanol, petroleum ether, ethyl acetate, dichloromethane and n-butanol were acquired from the General-Reagent (Shanghai Titan Scientific Co. Ltd., China). Malondialdehyde (MDA) content assay kit and superoxide dismutase (SOD) assay were bought from Jiancheng Bioengineering Institute (Nanjing, China). The Anti-α-SMA was purchased from Cell Signaling Technology (USA).

### Preparation of the ethyl acetate extract from *Ephedra* root

Roots of *Ephedra sinica* Stapf (ES) were collected from Inner Mongolia in 2022 and authenticated by Professor Haobin Hu in Jiangsu Institute for Food and Drug Control. The voucher specimen ((No. G20220908) was deposited in the authors’ laboratory (The Key Laboratory of the State Administration of Traditional Chinese Medicine). The dried roots of ES were grinded and passed through a 60-mesh sieve. The powder (220 g) was extracted twice under ultra-sonication at 60 °C and the obtained extract were filtered, dried, re-suspended in water and partitioned successively with petroleum ether, ethyl acetate and n-butanol. The ethyl acetate extract of *Ephedra* roots was obtained and stored at − 20 °C before use.

### Comprehensive chemical analysis of polyphenols in ERE using column chromatography and UPLC-LTQ-Orbitrap-HRMS

The ethyl acetate extract of *Ephedra* roots obtained in Sect. “Preparation of the ethyl acetate extract from Ephedra root” was subjected to normal-phase silica gel column chromatography (100–200 mesh) and eluted with a dichloromethane-methanol gradient (v/v 1:0 → 0:1) to yield eight fractions (Fr 1-Fr 8). Fr 5 was further separated by silica gel column chromatography using the same dichloromethane-methanol gradient system (v/v 1:0 → 0:1), yielding eight subfractions (Fr 5.1-Fr 5.8). The subfractions were then chromatographed over an ODS column with the methanol–water gradient system. Based on thin layer chromatography (TLC) and HPLC analysis, Fr 5.3.1, Fr 5.3.2, Fr 5.4.1, Fr 5.4.2, and Fr 5.5.2.1, designated as MH1-MH5 (Fig. S1), were found to be rich in PACs. They were further analyzed by UPLC-LTQ-Orbitrap-HRMS for tentative identification.

Chromatographic separation was performed on a UPLC system (Thermo Fisher Scientific) using the ACQUITY UPLC BEH C18 column (2.1 mm × 100 mm; 1.7 μm; Waters). The column temperature was set at 35 °C. The autosampler temperature was maintained at 4 °C and the injection volume was 5 μL. The mobile phase consisted of water with 0.1% formic acid (A) and methanol (B). The gradient elution program was as follows: the mobile phase B was increased to 10% within 0.5 min, followed by a linear increase to 30% within 9.5 min. Then the mobile phase B was increased to 50% within 10 min, 75% within 2 min and finally to 90% for another 4 min. The LTQ-Orbitrap Elite mass spectrometer was equipped with an electrospray ionization (ESI) source, which operated in the negative ion mode. The scan range for *m/z* (mass-to-charge ratio) was set at 50–1000. The collision energy was 35 eV, and the temperature of the capillary and heater block was 350 °C.

### SU5416/hypoxia-induced pulmonary hypertension rats and drug treatment

Male Sprague–Dawley rats (SPF grade, 6–8 weeks, weighing 180–200 g) were supplied by Weitong Lihua Laboratory Animal Technology Co., Ltd. (Zhejiang, China; production certificate SCXK 2020–0002). They were housed in a temperature (22–25 °C)-controlled environment with a 12 h light/dark cycle. Animals were monitored daily for signs of distress, including weight loss, lethargy, dyspnea, or reduced mobility. This study was conducted and reported in compliance with the ARRIVE (Animal Research: Reporting of In Vivo Experiments) guidelines. All experimental procedures were consented by the Institutional Animal Care and Use Committee of Yangzhou University and were conducted in strict accordance with the China Laboratory Regulation Act (2017) under a Project License (SYXK(SU)2017–0044).

**Fig. 1:**
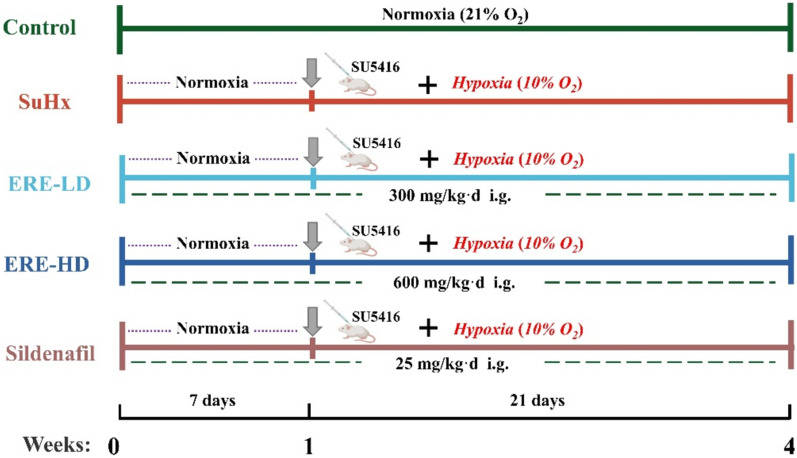
Schematic diagram of experimental design

Thirty rats were randomly divided into five groups, namely the normal control group (Control), the model group (SuHx), SuHx + ERE Low dose (ERE-LD), SuHx + ERE High dose (ERE-HD) and the SuHx + sildenafil (SD) positive control group. From day 1–7, rats in all groups were maintained in normoxia. From day 8, SuHx rats received a subcutaneous injection of SU5416 (20 mg/kg) and maintained in hypoxia for three weeks (10% O_2_). Rats in the control group received an equal volume of vehicle solution (5% DMSO, 5% Tween-80, 40% PEG300, 50% Saline) and kept in normoxia for three weeks. From day 1 to 28, rats in SuHx + ERE groups were treated with ERE (300 mg/kg and 600 mg/kg of ethyl acetate extract dissolved in 0.3% CMC-Na, respectively) once per day and rats in the SD positive control group were given SD (25 mg/kg) per day. In the meanwhile, rats in the control and model groups were treated with 0.3% CMC-Na. The doses of 300 mg/kg and 600 mg/kg for (ERE) were selected according to our previous published studies, preliminary experiment results and literature reference (Lv et al. 2022; Jin et al. 2016; Margalef et al. 2014). The experimental design was shown in Fig. 1.

### Echocardiography

After 3 weeks of hypoxia induction, SD rats were temporally anesthetized with 4% inhaled isoflurane, and then the concentration was adjusted to 2%, keeping their heart rate at 300–350 beats/min. The fur on the left chest was removed and rats were fixed on the heating pad (37 °C). Then, echocardiography was performed with an ultrasound system (IE33, Philip, Holland) to determine PAT and RVFW thickness.

### Measurement of hemodynamics and right ventricular hypertrophy

Hemodynamic data were measured by right heart catheterization. On the last day of the experiment, after intraperitoneal anesthesia with sodium pentobarbital (60 mg/kg), A polyethylene (PE) catheter filling with heparinized saline was inserted into the right jugular vein by cutting a V-shape incision and introduced into the right ventricle. The other end of the catheter was connected to a blood pressure sensor (Chengdu Techman Software, Chengdu, China) to determine the right ventricular systolic pressure (RVSP).

Rat hearts were harvested and dissected. The right ventricle (RV) and left ventricular septum (LV + S) were accurately weighed. RV hypertrophy was evaluated by the ratios of V to LV + S and RV to body weight.

### Histopathological examination

At the end of the experiment, major organs were harvested and weighed to calculate organ-to-body weight ratios (heart, lungs, liver, kidneys, spleen). Lung tissues were isolated and fixed in a 4% paraformaldehyde solution before being processed. Subsequently, they underwent dehydration with ethanol, xylene-based transparency, paraffin embedding, and were ultimately sectioned into 5 μm slices. Hematoxylin–eosin(H&E) staining and Masson’s trichrome staining were performed on these sections for morphological analysis. Pathological changes in pulmonary blood vessels were observed and photo-graphed under a microscope. Peripheral arterioles with a diameter ranging from 50 to 150 microns were selected for further analysis. ImageJ software was used to measure WT% (wall thickness percentage) and WA% (wall area percentage).

$$ {\text{WT}}\% \, = \,{2}\, \times \,{\text{WT }}/{\text{ ED}}\, \times \,{1}00\% $$

$$ {\text{WA}}\% \, = \,\left( {{\text{TA}} - {\text{VA}}} \right) \, /{\text{ TA}}\, \times \,{1}00\% $$

WT% represents the ratio of pulmonary arteriole wall thickness to the blood vessel diameter, while WA% indicates the proportion of pulmonary arteriole wall area to the total vascular area. WT refers to the thickness of the blood vessel wall; ED represents the outer diameter of the blood vessels; TA denotes the total vascular area; VA corresponds to the luminal area.

### Immunohistochemistry analysis

Paraffin-embedded lung tissue sections were dewaxed in xylene and dehydrated in alcohol, and hydrogen peroxide was added to block endogenous peroxidase. The sections were blocked with 5% bovine serum albumin (BSA) for 30 min, and then incubated with anti-α-SMA antibody overnight at 4 °C. On the next day, the secondary antibody (Goat Anti-Rabbit IgG (H + L)-HRP, Fcmacs, Nanjing, China) was added dropwise to the sections and incubated for 30 min. Then, SABC was added dropwise to cover the tissue and incubated in a 37 °C incubator for 20 min. DAB chromogenic agent was used to develop color for 2 min, and the color development was terminated by distilled water washing. Finally, the sections were stained with hematoxylin containing ethanol for 2 min, and the staining was terminated by distilled water washing after the time was up. Image J software was used to calculate the integral optical density (IOD) of α-SMA for each section, and statistical analysis was performed.

### Assay of oxidative stress levels

The lung tissue of rats and physiological saline were added to a centrifuge tube at a weight-to-volume ratio of 1:9, and homogenized thoroughly with an electric homogenizer. The homogenate was centrifuged using a low-temperature low-speed centrifuge, and the supernatant was taken. The levels of SOD and MDA in the supernatant were measured to reflect their levels in the lung tissue of rats. Detailed measurement steps were performed according to the kit instructions.

### Metabolomics analysis

#### Sample preparation

Blood was collected from the orbital venous plexus of rats. After allowing it to stand at room temperature for 2 h, the blood was centrifuged at 13,000 rpm at 4 °C for 10 min to obtain the supernatant, which was subsequently stored at −80 °C for future use. To each 20 μL serum sample, 100 μL of acetonitrile solution containing 2-chlorophenylalanine (5 μg/mL, internal standard) was added. The mixture was vortexed for 3 min and then centrifuged at 16,000 rpm at 4 °C for 10 min. The quality control sample was obtained by combining equal volumes of all serum samples, and its purpose was to monitor the reliability of the experiment.

#### Chromatography and mass spectrometry conditions

The untargeted metabolomic analysis was achieved by a combination of a UPLC system (Thermo Fisher Scientific) and an LTQ-Orbitrap Elite mass spectrometer (Thermo Fisher Scientific). Chromatographic separation was performed using the ACQUITY UPLC BEH C18 column (2.1 mm × 100 mm; 1.7 μm; Waters), and the column temperature was set at 35 °C, the autosampler temperature was maintained at 4 °C and the injection volume was 5 μL. The mobile phase consisted of water with 0.1% formic acid (A) and acetonitrile (B). The gradient elution program was as follows: from 0.1 to 1 min, the mobile phase B was increased to 10%, followed by a linear increase to 40% B within 2 min, then the mobile phase B was increased to 80% within 4 min, finally to 90% B within 4 min, and remained for another 4 min. The LTQ-Orbitrap Elite mass spectrometer was equipped with an electrospray ionization (ESI) source, which operated in both positive and negative ion modes. The scan range for *m/z* (mass-to-charge ratio) was set to 50–1000. The ion spray voltages in positive and negative modes were 3.8 kV and 3.2 kV, respectively. The capillary and heater temperature were both set to 350 °C.

### 16S rRNA gene sequence analysis

On the last day of the experiment, the feces were collected and stored at −80 °C. Qubit dsDNA Assay Kit was used to extract the total DNA. PCR using primer pairs 343F (5′-TACGGRAGGCAGCAG-3′) and 798R (5′-AGGGTATCTAATCCT-3′) was conducted to amplify the V3-V4 hypervariable region in the gene sequence of the bacterial 16S rRNA. The products were examined by agarose gel electrophoresis, purified using AMPure XP beads and quantified by Qubit. The sequencing was performed by Shanghai OE Biotech Co., Ltd. (Shanghai, China) with the Illumina NovaSeq 6000 platform. Subsequent alpha and beta diversity analysis were completed with the software QIIME 2. PICRUSt2 (Phylogenetic Investigation of Communities by Reconstruction of Unobserved States) was employed to predict the functional potential of the altered gut microbiota based on the 16S rRNA gene sequencing data.

### Statistical analysis

Data are expressed as mean ± standard deviation (SD). Differences between groups were analyzed using one-way analysis of variance (ANOVA). When the data did not follow a normal distribution, the Kruskal–Wallis rank sum test was used. Additionally, when the data were normally distributed but with unequal variances, Dunnett’s T3 multiple comparison test was employed. A *P*-value less than 0.05 was considered statistically significant. Data analysis and statistical graph plotting were performed using Graphpad Prism 8.0 software.

## Results

### Comprehensive chemical analysis of polyphenols in the ethyl acetate extract of *Ephedra* roots

The ethyl acetate extract from the roots of *Ephedra sinica* underwent silica gel and ODS column chromatography to afford 5 subfractions MH1-MH5. UPLC-LTQ-Orbitrap-HRMS analysis under the negative ionization mode was performed to characterize the polyphenols in MH1-MH5 and the detailed identification results was presented in Tables S1, S2, S3, S4, S5.

**Fig. 2:**
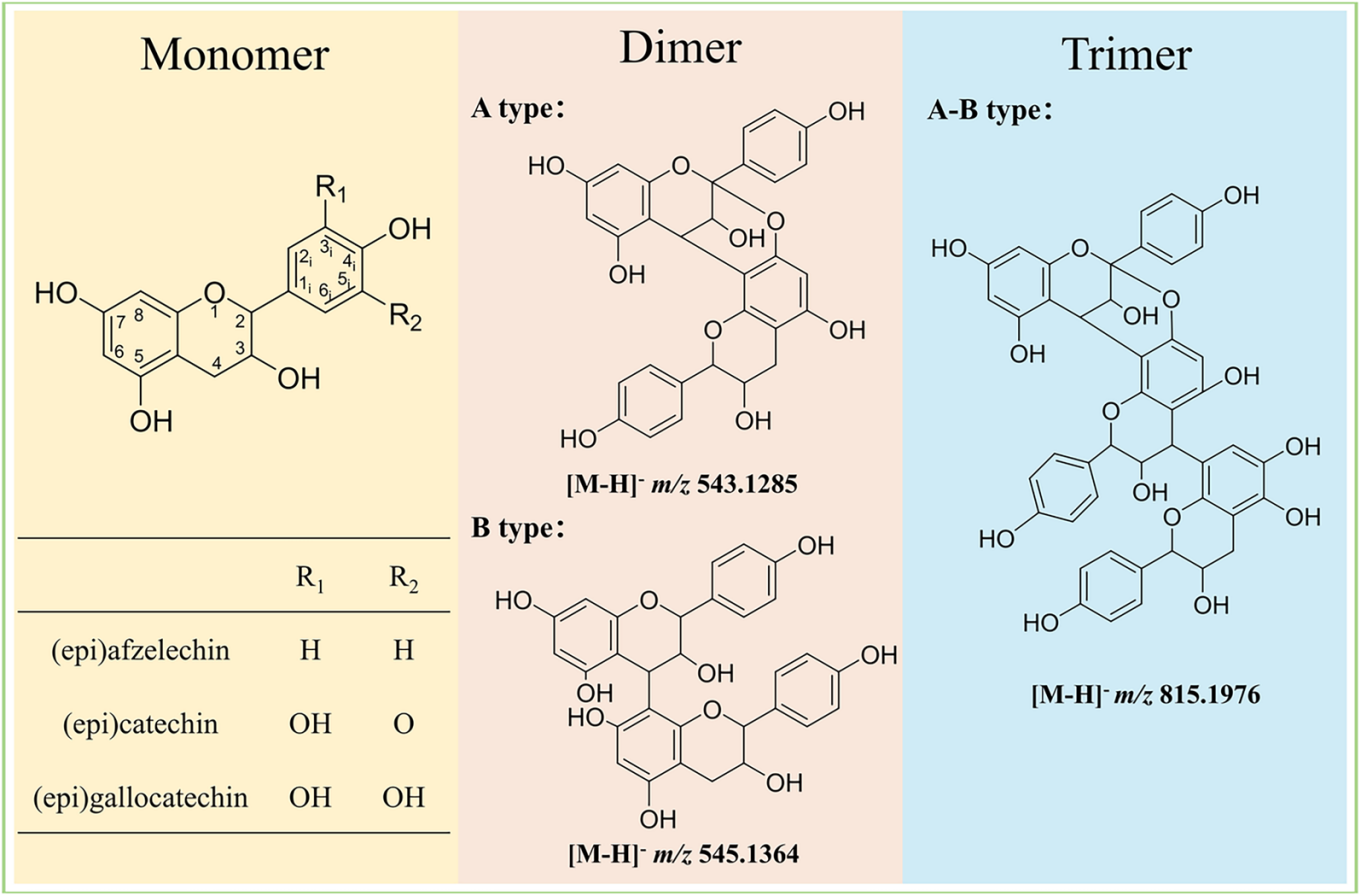
Tentative identification of proanthocyanidin monomers, dimers and trimers in the ethyl acetate extract of *Ephedra* roots

According to the diagnostic fragmentation patterns of precursor ions, proanthocyanidin monomers, dimers and trimers were tentatively identified in ERE (Fig. 2). Flavan-3-ols, mainly including (epi)afzelechin, (epi)catechin and (epi)gallocatechin, are the composing units of PACs, which can be classified based on the degree of polymerization, the monomeric units, and the types of interflavan linkages. Our previous findings mainly focused on the rarely reported A-type PAC dimers in ERE, however, after a two-step enrichment by column chromatography, the relative low levels of B-type PAC dimers and trimers were profiled. The representative fragmental mass spectra of A-type dimers and trimers were presented in supplementary Fig. S2. Product ions of Quinone Methide Fission (QM), Retro-Diels–Alder fission (RDA) and Heterocyclic ring fission (HRF) can be utilized to infer the linkage type and the composing units of PACs. The proposed fragmentation pathways for A-type and B-type dimers were shown in Figs. S3 and S4. They have a 2 Da difference in the *m/z* of [M-H]^−^ and corresponding product ions. For the tentative identification of trimers, the Peak [M-H]^−^ *m/z* 815.19 in MH5 was tentatively identified as (epi)afzelechin-B-(epi)afzelechin-A-(epi)afzelechin (Fig. S5), which could generate product ions of *m/z* 689.1676 and *m/z* 679.1443 due to a loss of 126 Da and 136 Da via HRF and RDA cleavage pathways, respectively. Besides, the ion at *m/z* 543.1299 indicated that the first QM cleavage occurred at the B-type bond, and it can be further fragmented through QM, RDA and HRF pathways to obtain products ions of *m/z* 269.0462, *m/z* 273.0773, *m/z* 407.0762 and *m/z* 417.0985.

### Effects of ERE on pulmonary artery pressure and right ventricular hypertrophy in SuHx rats

**Fig. 3:**
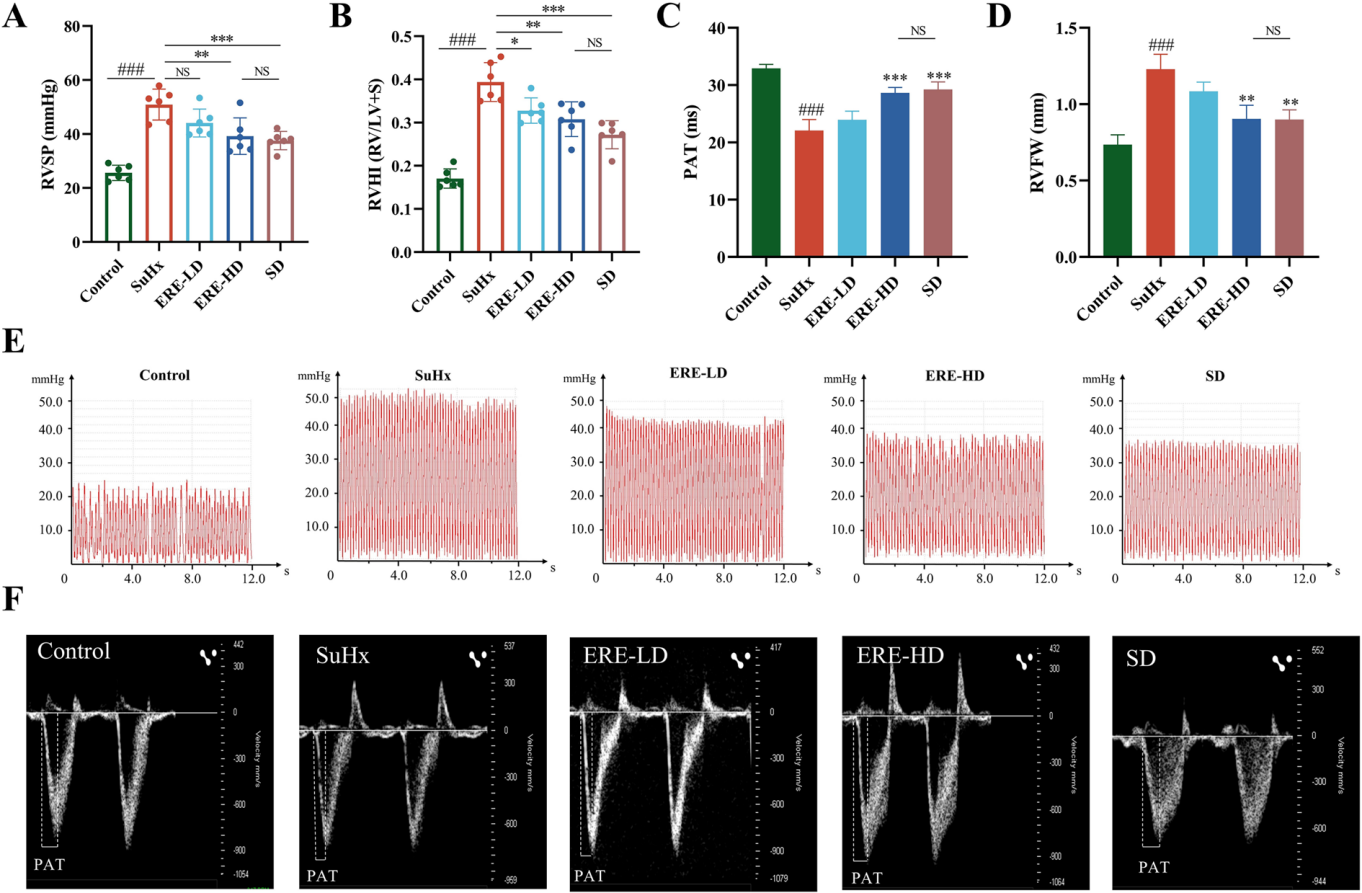
Effects of ERE on pulmonary hemodynamics in SuHx rats. RVSP (**A**) and RVHI (**B**) in different groups of rats; **C** pulmonary artery acceleration time in different groups of rats; **D** right ventricular free wall thickness in different groups of rats; **E** representative images of right ventricular pressure waveforms and ultrasound results (**F**) in rats of each group. All the data were expressed as mean ± SD (n = 6). ^###^*p* < 0.001 compared with Control group; *p < 0.05, **p < 0.01, ***p < 0.001 compared with SuHx group. NS represented no statistical significance

After a three-week period of SU5416 administration coupled with hypoxic conditioning, the pulmonary hemodynamic parameters of rats were measured. The RVSP of rats was assessed by right heart catheterization. As shown in Fig. 3A, RVSP of rats in the SuHx group was significantly higher than that in the control group, and a high dose of ERE could significantly inhibit the increase in RVSP (*P* < 0.01). Representative waveforms of right ventricular pressure for each group were presented (Fig. 3E). Regarding the right ventricular hypertrophy index (RVHI), data presented in Fig. 3B revealed that rats in the ERE group had a significantly lower RVHI than those in the SuHx group (*P* < 0.05). After three weeks of hypoxic treatment, SuHx rats displayed obvious symptoms, such as shortness of breath, cyanosis of the mouth and paws, lethargy, unkempt and dull hair, and fatigue. Rats in the ERE-LD group showed some improvement, while symptoms in the ERE-HD and SD groups were significantly delayed and alleviated compared to the SuHx rats. Ultrasound results, as shown in Fig. 3C, indicated that the PAT was considerably reduced after the modeling process, but improved in drug administration group. Remarkable differences were observed between the ERE-HD and SD groups when compared to the model group (*P* < 0.001). Moreover, compared with the Control group, the RVFW of SuHx rats increased notably (Fig. 3D), and both a high dose of ERE and the positive control drug could significantly inhibit the increase in wall thickness (*P* < 0.01). Representative ultrasound images for each group were displayed in Fig. 3F. Collectively, these findings suggested that ERE exhibited a significant inhibitory effect on the increase of RVSP, RVHI, the decrease in PAT, and the rise in RVFW in rats with pulmonary hypertension induced by the combination of SU5416 and hypoxia.

### Effects of ERE on SU5416/hypoxia-induced pulmonary vascular remodeling

**Fig. 4:**
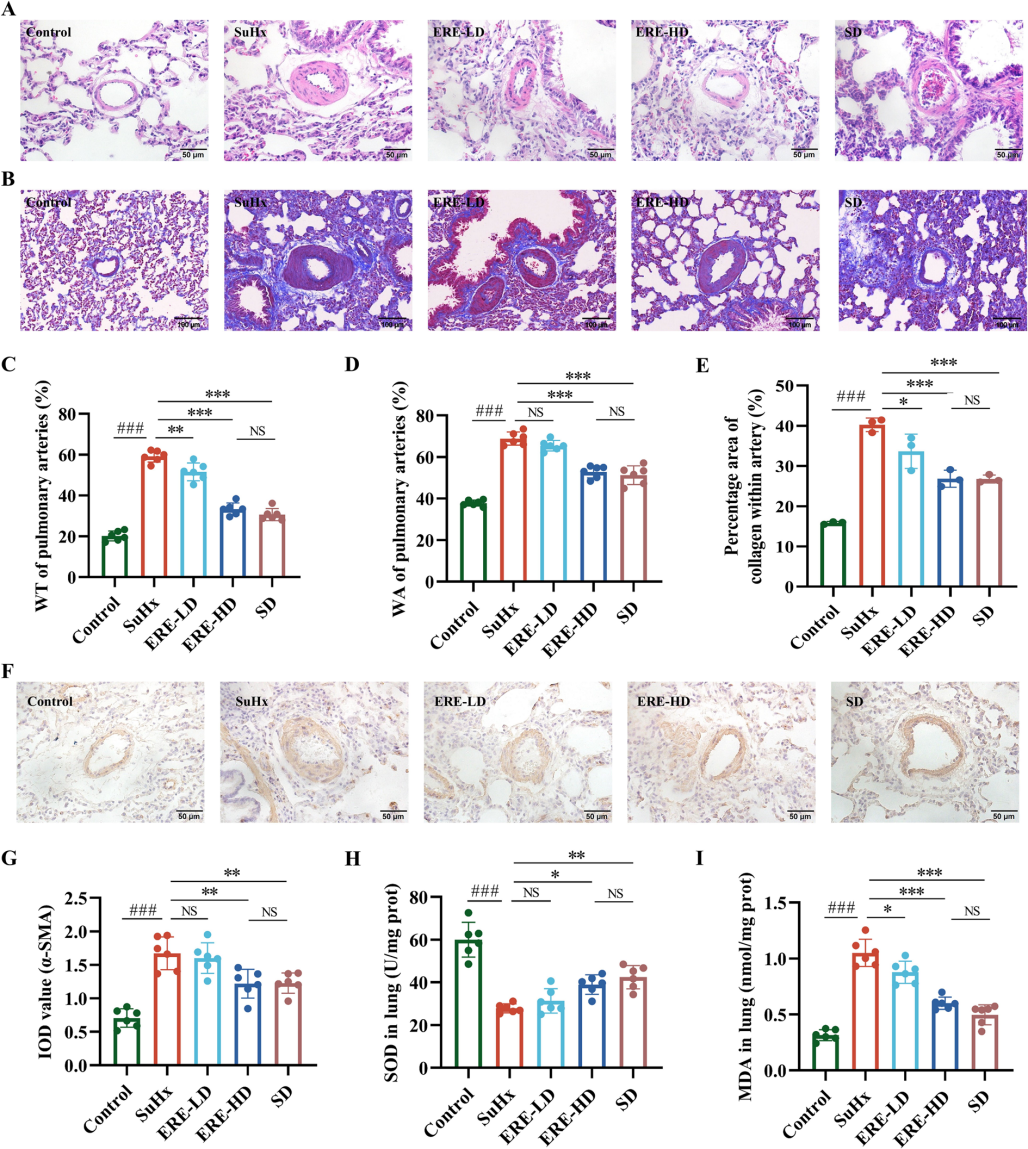
Effects of ERE on pulmonary vascular remodeling in SuHx rats. Representative images of H&E staining (**A**) and Masson staining (**B**) in the lung tissues of rats; The related indexes of pulmonary vascular remodeling, WT% (**C**) and WA% (**D**), were measured; **E** The percentage area of collagen within artery; **F** Representative images of immunohistochemical staining in the lung tissues of rats and integral optical density of α-SMA (**G**) in different groups; Levels of SOD (H) and MDA (**I**) in rat lung tissue. All the data were expressed as mean ± SD (n = 6). ^###^*p* < 0.001 compared with Control group; *p < 0.05, **p < 0.01, ***p < 0.001 compared with SuHx group. NS represented no statistical significance

Pulmonary vascular remodeling plays a pivotal role in the development of PH. To evaluate the pulmonary artery remodeling, H&E staining and Masson trichrome staining were conducted to detect the pulmonary arteriole wall thickness. As showed in Fig. 4A, the walls of small pulmonary arteries were thin and regular in the Control group, whereas in the SuHx group, the arterial walls were markedly thickened and irregular, with evidence of intimal necrosis and denudation, medial hypertrophy, and lumen narrowing. These pathological changes were significantly relieved following ERE treatment. A significant increase in the WT% and WA% was observed in the SuHx group when compared with the those in the Control group. Treatment with sildenafil and HD-ERE markedly down-regulated the SU5416/hypoxia-induced increase of WT% and WA% (Fig. 4C and D). Pulmonary vascular fibrosis is characterized by excessive collagen deposition in arterioles, which is exacerbated by SU5416/hypoxia exposure. Treatment with sildenafil, LD-ERE and HD-ERE greatly relieved deposition of collagen fibers, indicating that ERE could mitigate SU5416/hypoxia induced pulmonary artery fibrosis (Fig. 4B and E). The organ-to weight ratios (heart, lungs, liver, kidneys, spleen) among different experimental groups (Fig. S6) showed that no significant alterations indicative of organ-specific toxicity from ERE treatment and ERE treatment rescued the SU5416/hypoxia-induced elevation of heart-to-body and lung-to-body weight ratios.

### Effects of ERE on SU5416/hypoxia-induced pulmonary arteriomyosis and lung oxidative stress levels

SU5416/hypoxia induced pulmonary arteriomyosis, which was alleviated by ERE treatment (Fig. 4F and G). Results of the immunohistochemical analysis revealed that the IOD value of α-SMA markedly increased in the SuHx group, while ERE at high dosage significantly reduced the elevation. As indicators of oxidative stress in lung tissue, the levels of MDA and SOD were assessed in the lung tissue of rats from each group. Compared with the Control group, the MDA level in the lung tissue of rats in the SuHx group was significantly increased, and the SOD level was significantly decreased (Fig. 4H and I). It is worth noting that all ERE dose groups exhibit substantially lower MDA levels than the model group (*P* < 0.05), and specifically, the ERE-HD group boasts a significantly higher SOD level than the SuHx group (*P* < 0.01).

### Effects of ERE on serum metabolites in PH rats

**Fig. 5:**
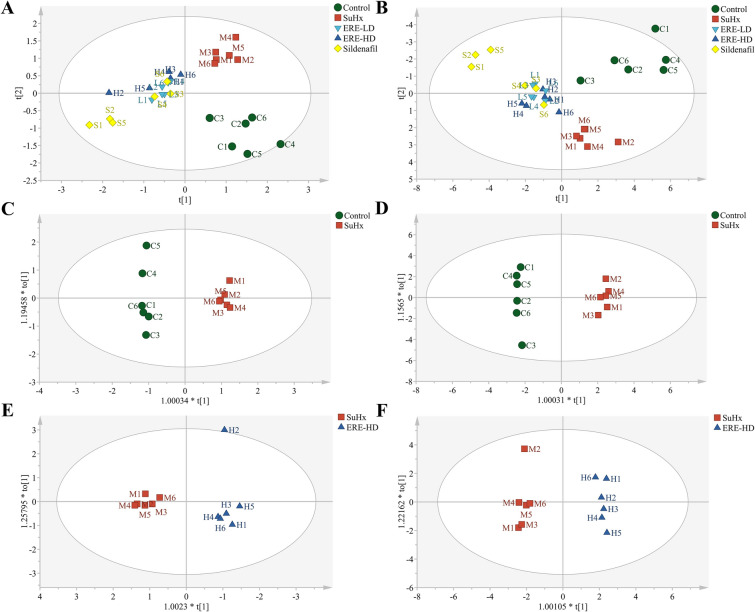
Effects of SuHx modeling and ERE intervention on serum metabolites in rats. **A**, **B** The metabolic profile for partial least squares discriminant analysis (PLS-DA) score scatter plots of serum samples (A: ESI ( +): R^2^X = 0.747, R^2^Y = 0.957, Q^2^ = 0.528; B: ESI (-): R^2^X = 0.596, R^2^Y = 0.832, Q^2^ = 0.528); **C**–**F** OPLS-DA models (Control vs. SuHx; SuHx vs. ERE-HD) for pairwise comparison

To capture the serum metabolic changes in ERE-treated PH rats, UPLC-LTQ-Orbitrap-based untargeted metabolomics was performed. The raw LC–MS data in both positive and negative ionization modes were subject to multivariate statistical analysis using SIMCA-P after pretreatment. The clustered QC samples in the PCA plots of all samples (Fig. S7) indicated the reliability and reproducibility of the bioanalytical method. Then the supervised PLS-DA was conducted to visualize the metabolic shifts induced by SU5416/hypoxia exposure and ERE treatment. As shown in Fig. 5A and B, clear separation was observed between the metabolic profiles of the SuHx and Control groups. Notably, ERE or sildenafil treatment significantly shifted the metabolic profiles induced by SU5416/hypoxia exposure, then pairwise comparison of OPLS-DA models (Control vs. SuHx; SuHx vs. ERE-HD; Fig. 5C, D, E, F) were constructed to identify differential metabolites that contributing to group separation and the parameters of different models were provided in Table S6.

**Fig. 6:**
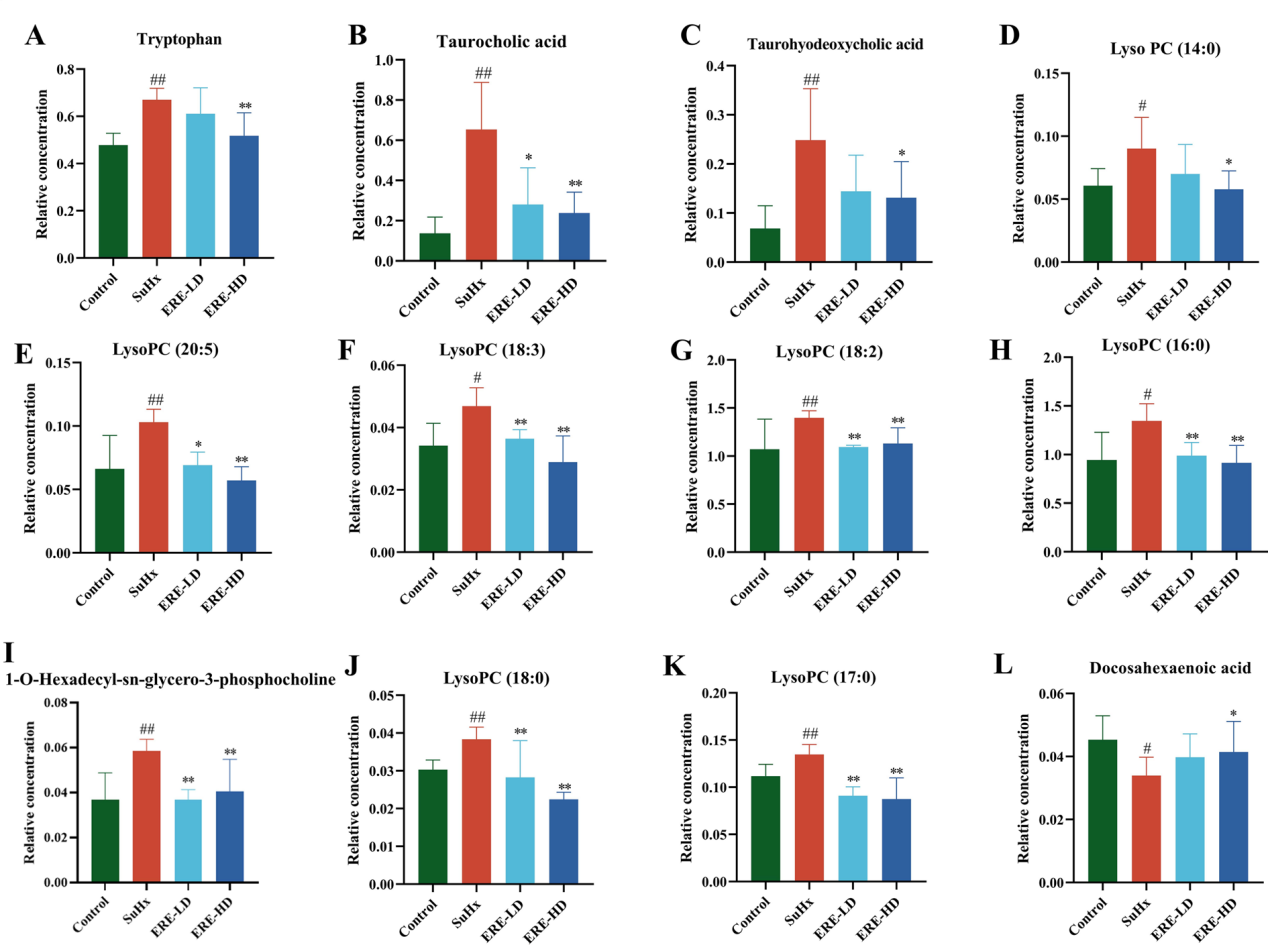
The relative concentration levels of differential metabolites that related to the therapeutic effects of ERE on SU5416/hypoxia-induced PH rats. ^#^*p* < 0.05, ^##^*p* < 0.01 compared with Control group; *p < 0.05, **p < 0.01, compared with SuHx group

VIP values (VIP > 1) obtained from OPLS-DA models and* P* values (*P* < 0.05) from Mann–Whitney test were used to screen differential variables, which were kept for structural annotation. After comparison of the precursor and diagnostic fragment ion information with the household and public databases such as the HMDB (https://hmdb.ca) and Metlin (http://metlin.scripps.edu), the levels of 12 metabolites involving glycerophospholipid, bile acid and tryptophan metabolism were significantly reversed through ERE treatment (Fig. 6 and Table S7).

### Effects of ERE on alpha and beta diversity of gut microbiota in PH rats

**Fig. 7:**
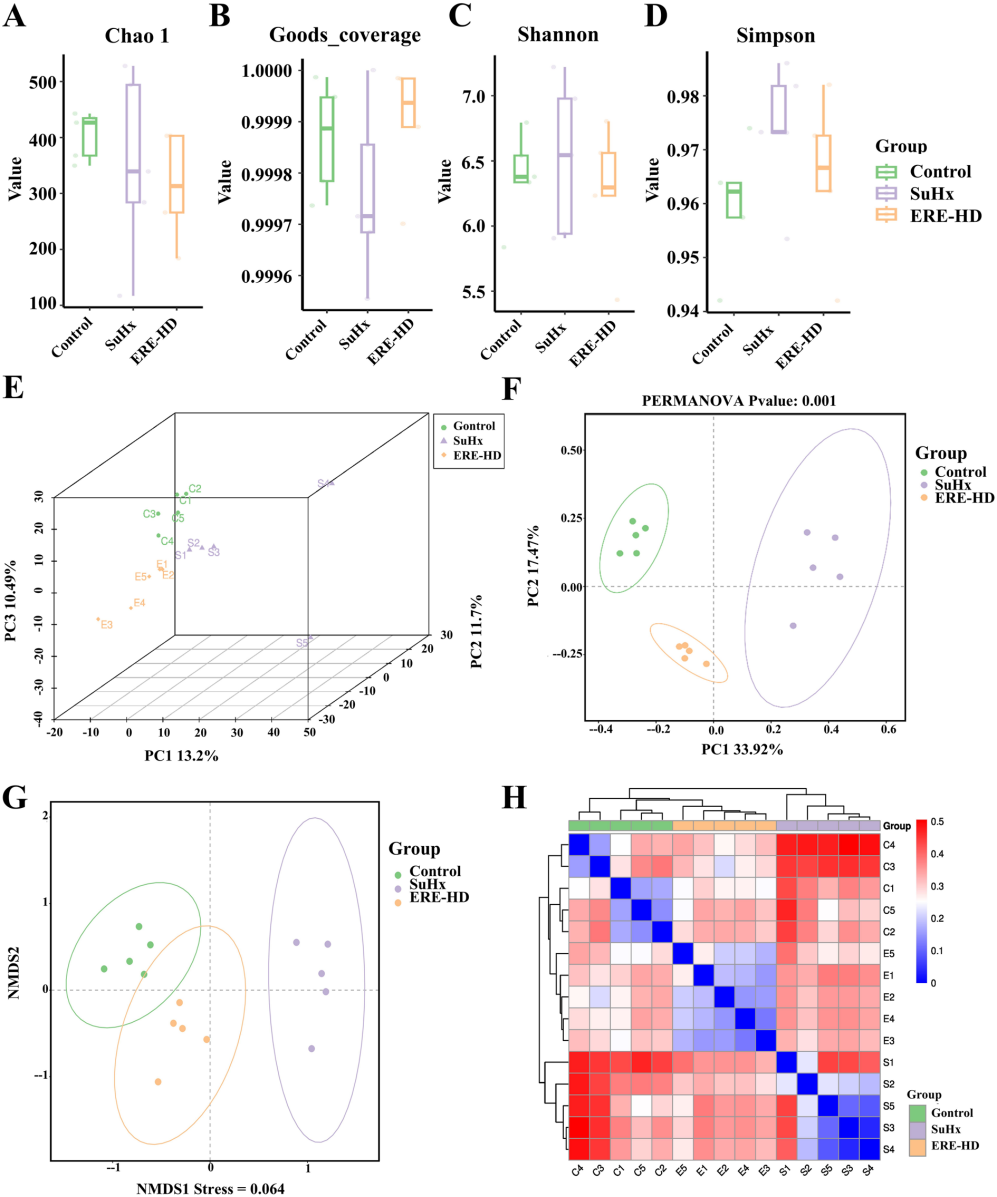
Effect of ERE on α and β diversity of intestinal microbiota in SuHx rats. The Chao 1 (**A**), Good’s Coverage (**B**), Shannon (**C**) and Simpson (**D**) indices of all samples (n = 5); Combined plot integrated with the PCA (**E**), PCoA (**F**), NMDS (**G**) and heatmaps (**H**) of the bacterial communities corresponding to beta diversity. All data are presented as the mean ± SD (n = 5)

The compositional alterations of intestinal microbiota in SuHx model rats following ERE intervention were analyzed via 16S ribosomal RNA (16S rRNA) gene sequencing. Alpha diversity, assessed using the Chao1 index, Good’s coverage, Shannon index, and Simpson index (Fig. 7A, B, C, D), revealed no significant intergroup differences. These findings suggest that neither the modeling process nor pharmacological intervention exerted a measurable impact on the overall richness or diversity of the gut microbial community. Notably, the Good’s coverage values across all experimental groups exceeded 0.999, with no statistically significant variations observed, confirming that sequencing depth adequately captured > 99.9% of the microbial diversity present in the fecal samples.

Beta diversity, evaluated through principal component analysis (PCA), non-metric multidimensional scaling (NMDS), and principal coordinate analysis (PCoA) (Fig. 7E, F, G, H), demonstrated a pronounced separation in microbial community structure among experimental groups. Results show that, the ERE-HD cohort exhibited closer clustering to the Control group relative to the SuHx model group, suggesting a partial normalization of intestinal microbiota composition following high-dose ERE treatment.

### Effects of ERE on gut microbiota changes at the phylum and family level in PH rats

**Fig. 8:**
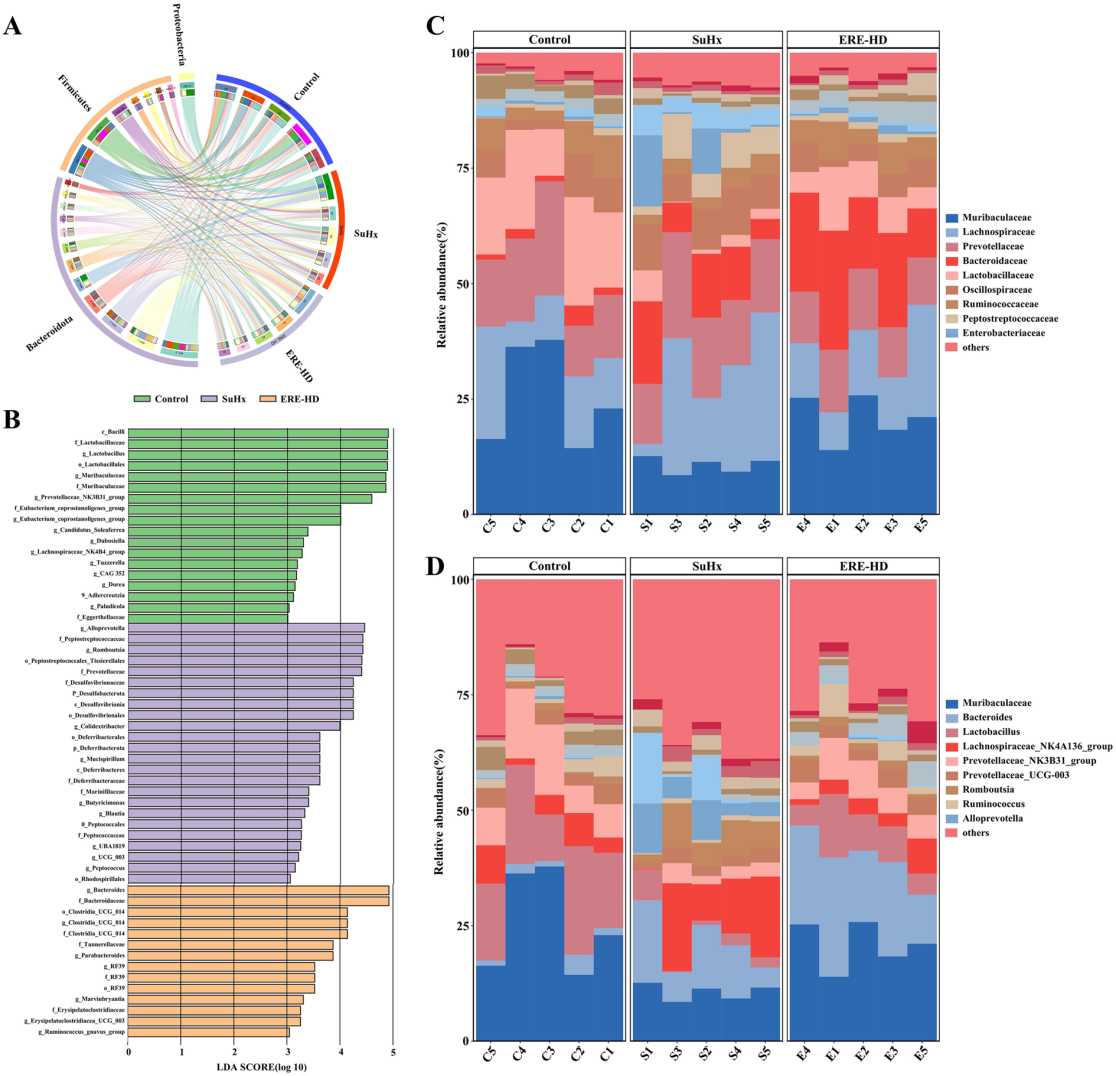
The effect of ERE on the gut microbiota of SU5416/hypoxia-induced PH rats. **A** Bacterial taxonomic characteristics of the intestinal microbiota at the phylum level; **B** The distribution map of intestinal microbiota with LDA scores exceeding 3 in the three groups of rats; **C** The relative abundance of bacterial communities in different groups at the family level; **D** The relative abundance of bacterial communities in different groups at the genus level

At the phylum level, the Circos plot (Fig. 8A) revealed the relationships between different ASVs and experimental groups. *Bacteroidota*, *Firmicutes* and *Proteobacteria* are the predominant bacterial phyla, accounting for more 90% of all microbial taxa. Linear discriminant analysis with effect size (LEfSe) analysis was conducted to identify significantly different microbial taxa among the three groups of rats, aiming to uncover potentially pathogenic bacteria enriched in the SuHx group and beneficial bacteria enriched in the ERE-HD group. As shown in Fig. 8B, when the linear Discriminant analysis (LDA) score threshold was set at > 3, 31 taxa were screened out from phylum to genus. Significantly enriched taxa for the control group were *Bacilli*, *Lactobacillaceae*, *Lactobacillus*, *Lactobacillales*, *Muribaculaceae*, etc. For the SuHx group, the taxa such as *Alloprevotella*, *Peptostreptococcaceae*, *Romboutsia*, *Peptostreptococcales_Tissierellales* and *Prevotellaceae* were significantly higher in abundance. However, for the ERE-HD group, *Bacteroides*, *Bacteroidaceae* and *Clostridia_UCG_014* exhibited marked difference in abundance.

**Fig. 9:**
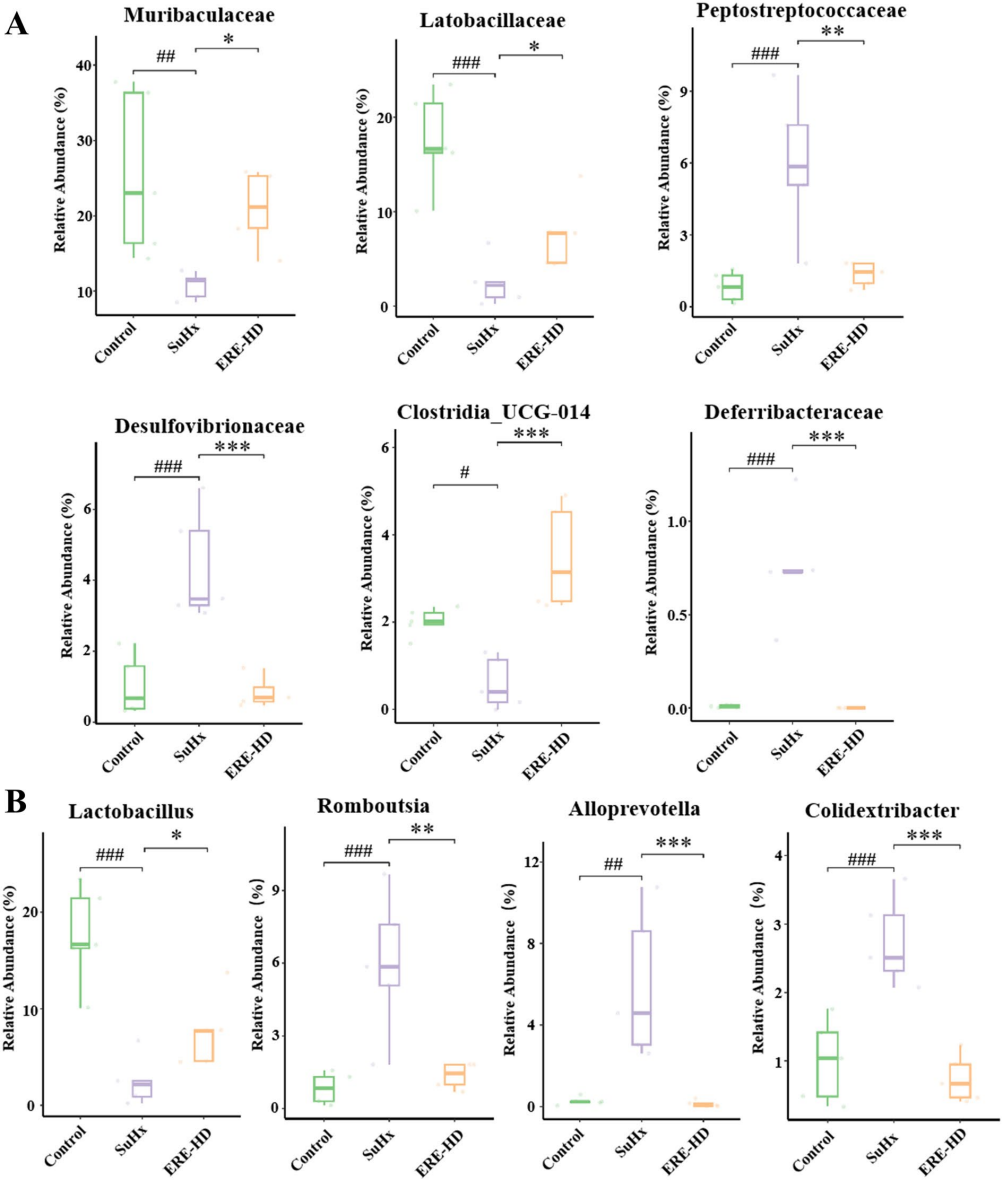
**A** The relative abundance of differential intestinal microbial family of *Muribaculaceae*, *Latobacillaceae*, *Peptostreptococcaceae*, *Desulfovibrionaceae*, *Clostridia_UCG-014* and *Deferribacteraceae*; **B** The relative abundance of differential intestinal microbial genus including *Lactobacillus*, *Romboutsia*, *Alloprevotella* and *Colidextribacter* in three groups; The *p* values were defined as follows: ^#^*P* < 0.05, ^##^*P* < 0.01, ^###^*P* < 0.001(“^#^” represents a significant difference comparing with the Control group); *P < 0.05, **P < 0.01, ***P < 0.001 (“*” represents a significant difference comparing with the SuHx group)

The relative abundance of major microbial taxa across all experiment groups at the family and genus level were presented in Fig. 8C and D. As to the family level (Fig. 9A), ERE significantly reduces the up-regulated level of *Peptostreptococcaceae*, *Desulfovibrionaceae* and *Deferribacteraceae*, and recovered the decreased relative abundance of *Muribaculaceae*, *Latobacillaceae* and *Clostridia_UCG—014* induced by SU5416/hypoxia exposure. At the genus level (Fig. 9B), the ERE treatment remarkably reversed the changes in the level of *Lactobacillu*, *Romboutsia*, *Alloprevotella* and *Colidextribacter*. The functional potential of the altered gut microbiota was visualized in supplementary Fig. S8. Additionally, an integrative correlation analysis between the significantly altered gut microbiota and the significantly changed metabolites was performed and presented in supplementary Fig. S9.

## Discussion

Emerging pharmacologic evidence demonstrated the role of metabolic and gut microbial dysregulation in the pathogenesis of pulmonary hypertension (PH), unveiling novel therapeutic targets. This paradigm shift supports the strategic application of multi-targeted natural products (Wang et al. 2024b; Zhang et al. 2024). In our study, column chromatography combined with UPLC-LTQ-Orbitrap-HRMS analysis revealed that ERE mainly contained A-type PAC dimers and a few monomers and trimers. Notably, the most common composing unit of PACs from ERE was (epi)afzelechin, which was different from those in grape seed. It is reported that epiafzelechin and ephedrannin A isolated from the *Ephedra* roots could significantly decrease the systolic and diastolic pressure of spontaneously hypertensive rats (SHR) (Yang et al. 2010). However, their impact on PH remains unknown.

The SU5416/hypoxia (SuHx)-induced PH rat model, which combines the injection of a vascular endothelial growth factor receptor (VEGFR) inhibitor SU5416 with chronic exposure to hypoxia, is widely regarded as one of the most robust and clinically relevant experimental models for PH (Boucherat et al. 2022; Chen et al. 2020; Hong et al. 2021). One of the key pathological features in PH is the vasoconstriction and vascular remodeling, which contributes to elevated pulmonary arterial pressure and RV hypertrophy (Naeije et al. 2022). In the present study, we demonstrated for the first time that ERE significantly ameliorated the pathological and functional features of PH in the SU5416/hypoxia (SuHx)-induced rat model. Oxidative stress has been increasingly recognized as an important contributor to PH pathogenesis, where excessive production of reactive oxygen species (ROS) leads to endothelial injury, PASMC proliferation, and inflammation (Pokharel et al. 2023; Xu et al. 2022). In this context, antioxidant defense mechanisms, particularly SOD, play a crucial role in maintaining redox homeostasis and vascular integrity (Poyatos et al. 2023). Conversely, elevated levels of MDA, a byproduct of lipid peroxidation, serve as a reliable marker of oxidative tissue damage (Lankin et al. 2022). Our findings are consistent with previous reports that polyphenol-rich plant extracts can modulate antioxidant enzyme activity and suppress oxidative stress in models of cardiovascular and pulmonary injury (Alotaibi et al. 2021; Rudrapal et al. 2024).

Although PACs exhibited low bioavailability, previous in vitro and in vivo research reported that PACs with a degree of polymerization less than 5 are absorbable, and they can be extensively metabolized by the gut microbiota into highly bioavailable, low-molecular-weight phenolic acids (Ou et al. 2014). Besides, there is growing evidence for the “gut-lung axis,” where gut-derived metabolites and immune signals can profoundly influence systemic inflammation and remote organ health, including the lungs (Yang et al. 2025). The modulation of gut microbiota and the subsequent release of anti-inflammatory metabolites or the influence on immune cell priming is a potential mechanism that may explain the strong systemic effects of compounds with relatively low bioavailability (Wang et al. 2025). Therefore, it’s imperative to elucidate the pharmacological mechanism of PACs from the perspective of gut microbial modulation and host metabolism.

Our metabolomics study showed a significant increase of lysophosphatidylcholine (LysoPC) levels in PH rats, which corresponded with the increased vascular and pulmonary inflammation in PH patients. However, ERE-HD significantly reduced the LysoPC levels, suggesting a corrective effect on glycerophospholipid metabolic imbalance. LysoPC is a hydrolytic product of phosphatidylcholine (PC), and it belongs to a category of bioactive phosphatidylcholine derivatives with various physiological functions. Within the cardiovascular system, LysoPC can exert cytotoxic effects on vascular endothelial cells through upregulating the expression of inflammatory factors and inducing elevated cellular oxidative stress levels (Chang et al. 2017; Li et al. 2015), thus being regarded as a pro-inflammatory lipid molecule (Li et al. 2018). Conversely, docosahexaenoic acid (DHA), a distinctive ω-3 series polyunsaturated fatty acid that exerts anti-inflammatory, antiplatelet activation, and vascular-protective properties, was significantly reduced in the SuHx group (*P* < 0.05) and was restored following ERE administration (*P* < 0.05), highlighting ERE’s potential to restore protective lipid mediators (Chen et al. 2017; Nagaraj et al. 2016). Additionally, the levels of tryptophan, taurocholic acid (TCA), and taurohyodeoxycholic acid (THDCA) were significantly reversed in PH rats following ERE treatment. These metabolites are known to influence inflammation, immune responses, and gut microbial composition, all of which are implicated in PAH progression, which requires targeted metabolomics study in the future (Jasiewicz et al. 2016; Li et al. 2023; Teunis et al. 2023).

In addition to metabolic modulation, ERE exhibited a profound influence on gut microbial composition, which is increasingly recognized as a critical factor in cardiopulmonary health. *Muribaculaceae* is known for producing beneficial metabolites such as short-chain fatty acids (SCFAs), which are reported to modulate the onset and progression of PH through multiple mechanisms. Reduced SCFA production compromises gut barrier integrity and increases intestinal permeability, facilitating the translocation of harmful microbial-derived metabolites into the bloodstream, which may contribute to systemic inflammation and vascular dysfunction (Kim et al. 2020). SCFAs have been reported to attenuate hypoxia-induced pulmonary vascular remodeling by regulating cytokine production in lung tissue (Karoor et al. 2021; Park et al. 2015). Endothelial dysfunction is recognized as a critical contributor to the pathogenesis of PAH. *Lactobacillaceae* may preserve endothelial cell function either by secreting bioactive metabolites or through direct modulation of endothelial signaling pathways (Yan et al. 2022). Our integrative multi-omics analysis revealed a statistically significant negative correlation between host circulating tryptophan levels and the relative abundance of the *Lactobacillaceae* family in the gut microbiota of rats. It has been demonstrated that dysregulated gut microbiota may contribute to PH pathogenesis by disrupting tryptophan metabolism and aryl hydrocarbon receptor (AhR) signaling pathways (Yang et al. 2025). PH patients were reported to have higher blood concentrations of tryptophan (Wang et al. 2010). The tryptophan level exhibited a positive correlation with *Peptostreptococcaceae*, *Desulfovibrionaceae*, *Romboutsia* and *Alloprevotella* in our study. *Peptostreptococcaceae*, frequently associated with dysbiosis and various diseases, may exhibit pathogenic potential by activating the host’s intestinal immune system, leading to the overproduction of pro-inflammatory cytokines, such as tumor necrosis factor-alpha (TNF-α) and interleukin-6 (IL-6) (Wang et al. 2021a, b). These cytokines have been shown to play critical roles in the pathogenesis of PH by stimulating PASMC proliferation and contraction, thereby contributing to elevated pulmonary arterial pressure and vascular remodeling (Hu et al. 2022; Wang et al. 2021a, b; Xu et al. 2024). Future studies could involve fecal microbiota transplantation (FMT) to further prove the role of gut microbiota regulation in the therapeutic effects of ERE on PH rats.

## Conclusions

In the present study, polyphenols in the ethyl acetate extract of *Ephedra* roots were comprehensively characterized through column chromatography and UPLC-LTQ-Orbitrap analysis for the first time. Notably, ERE contained the rarely reported A-type proanthocyanidin dimers and trimer. Considering their relatively low oral bioavailability and good pharmacological effects, the impact of ERE on SU5416/hypoxia-induced pulmonary arterial hypertension rats was investigated through its interaction with gut microbiota and host metabolism. Oral administration of ERE significantly relieved PH symptoms such as the elevated pulmonary arterial pressure, right ventricular hypertrophy, thickened RVFW, increased PAT and pulmonary vascular remodeling. Collectively, ERE was proved to be a promising anti-PH agent through improving gut microbiota dysbiosis and the altered glycerophospholipid and amino acid metabolism. Our study not only laid scientific foundation for the better utilization of various proanthocyanidin monomers, dimers and trimers in *Ephedra* roots but also gave novel insights into the pharmacological study of polyphenols with low-bioavailability and high-efficacy.

## Supplementary Information

Additional file1 (DOCX 12142 KB)

## Data Availability

All data generated or analyzed during this study are included in this published article.

## Abbreviations

PACs: Proanthocyanidins
ERE: Ethyl acetate extract from the roots of Ephedra sinica Stapf
PAH: Pulmonary arterial hypertension
PH: Pulmonary hypertension
RVFW: Right ventricular free wall
PAT: Pulmonary acceleration time
COPD: Chronic obstructive pulmonary disease
MDA: Malondialdehyde
SOD: Superoxide dismutase
PE: Polyethylene
RVSP: Right ventricular systolic pressure
RV: Right ventricle
LV+S: Left ventricular septum
WT%: Wall thickness percentage
WA%: Wall area percentage
BSA: Bovine serum albumin
IOD: Integral optical density
ES: Ephedra sinica Stapf
TLC: Thin layer chromatography
Control: Control group
SuHx: Model group
ERE-LD: SuHx+ERE Low dose
ERE-HD: SuHx+ERE High dose
SD: Sildenafil
ESI: Electrospray ionization
RSD: Relative standard deviation
PCA: Principal components analysis
OPLS-DA: Orthogonal partial least squares discriminant analysis
VIP: Variable importance in projection
HMDB: Human Metabolome Database
KEGG: Kyoto Encyclopedia of Genes and Genomes
SD: Standard deviation
ANOVA: Analysis of variance
QM: Quinone Methide Fission
RDA: Retro-Diels-Alder fission
HRF: Heterocyclic ring fission
RVHI: Right ventricular hypertrophy index
H&E: Hematoxylin-eosin
NMDS: Non metric multidimensional scaling
PCoA: Principal coordinate analysis
LEfSe: Linear discriminant analysis with effect size
LDA: Linear discriminant analysis
SHR: Spontaneously hypertensive rats
VEGFR: Vascular endothelial growth factor receptor
ROS: Reactive oxygen species
LysoPC: Lysophosphatidylcholine
PC: Phosphatidylcholine
DHA: Docosahexaenoic acid
TCA: Taurocholic acid
THDCA: Taurohyodeoxycholic acid
SCFAs: Short-chain fatty acids
TNF-α: Tumor necrosis factor-alpha
IL-6: Interleukin-6
PASMC: Pulmonary arterial smooth muscle cell
