# Supplementary material for: Comprehensive chemical analysis of polyphenols in the ethyl acetate extract from the roots of Ephedra sinica Stapf and evaluation of its therapeutic effects on SU5416/hypoxia-induced pulmonary arterial hypertension rats
Source: Bioresour Bioprocess. 2025 Oct 29;12(1):120. doi: 10.1186/s40643-025-00963-9 (PMC12572481; doi:10.1186/s40643-025-00963-9)
Supplement: Supplementary file 1 — Additional file1 (DOCX 12142 KB) [file 40643_2025_963_MOESM1_ESM.docx]

**Comprehensive chemical analysis of polyphenols in the ethyl acetate extract from the roots of *Ephedra sinica* Stapf and evaluation of its therapeutic effects on SU5416/hypoxia-induced pulmonary arterial hypertension rats**

Mengying Lv ^1,2,3*^, Jinhao Shuai ^1,2^, Yang Wang ^1,2^, Qianwen Lu ^1,2^, Xinlong He ^1,2^, Junjie Zhen ^1,2^, Ling Ling ^1^, Jun Yao ^4,5*^, Fengguo Xu ^6*^

**
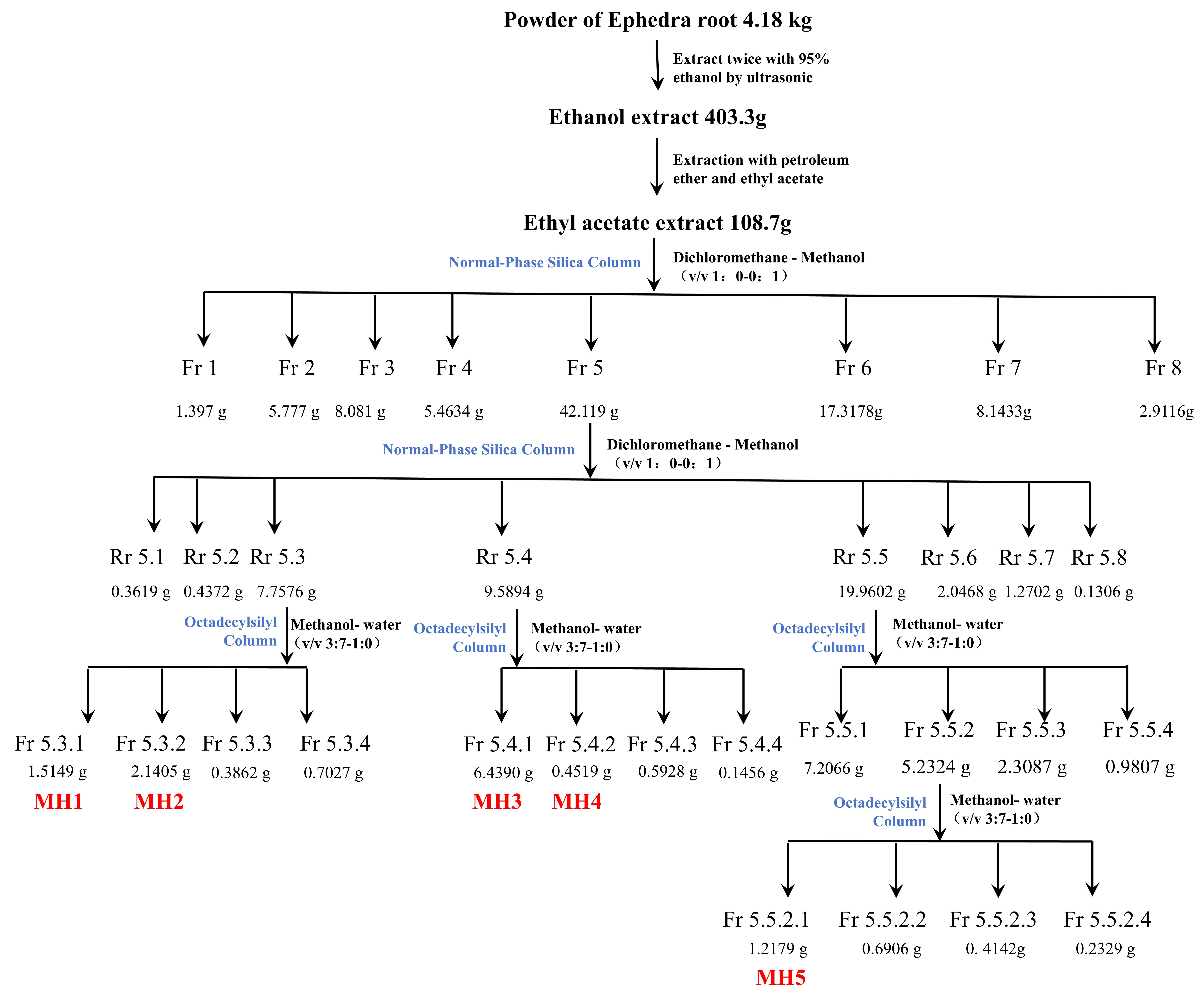
**

**Figure S1.** Flowchart of fractionation of the ethyl acetate extract of *Ephedra* root





**Figure S2. The representative fragmental mass spectra of A-type dimers and trimers**

**
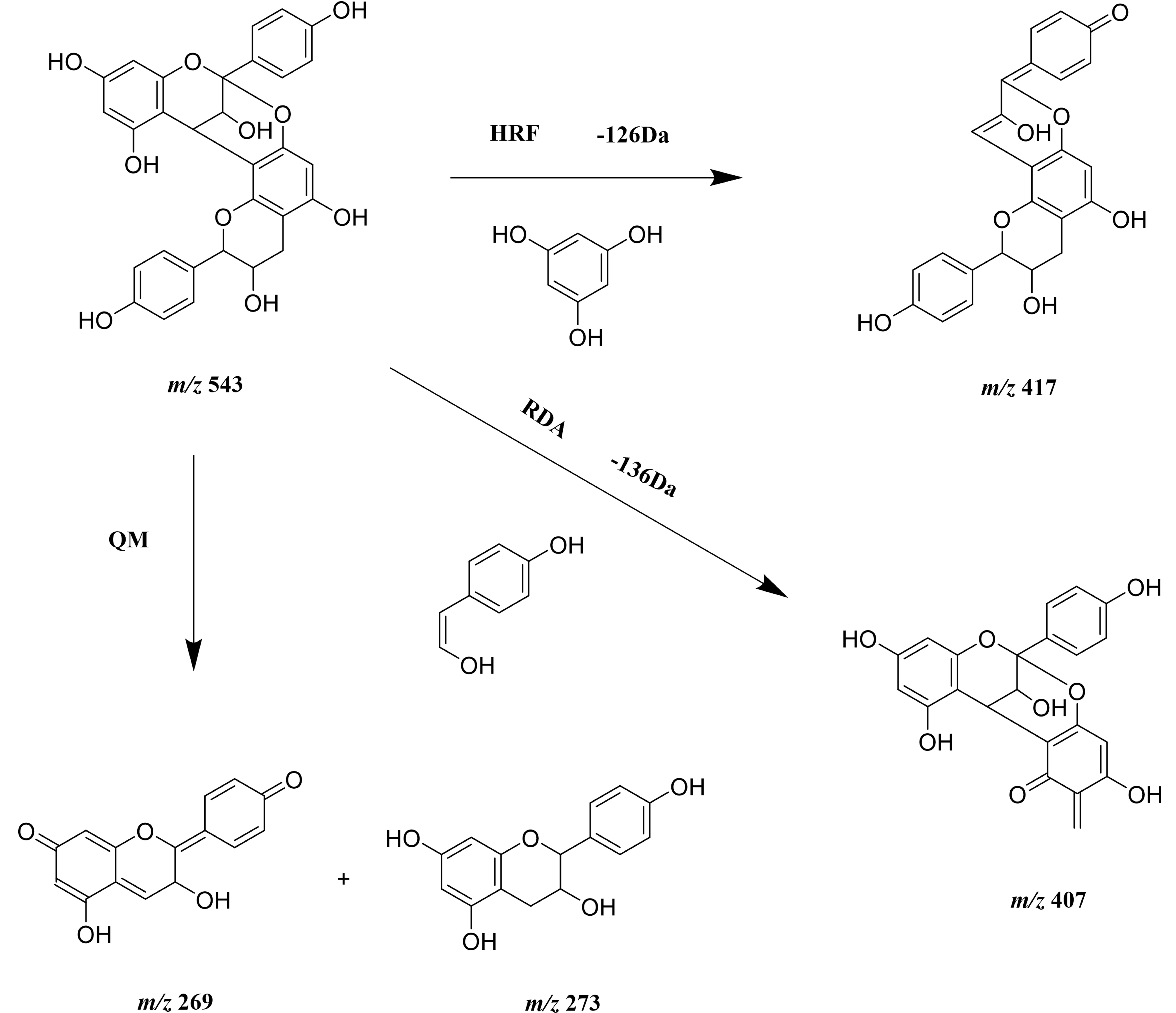
**

**Figure S3.** The potential fragmentation pathway of (epi)afzelechin-A-(epi)afzelechin


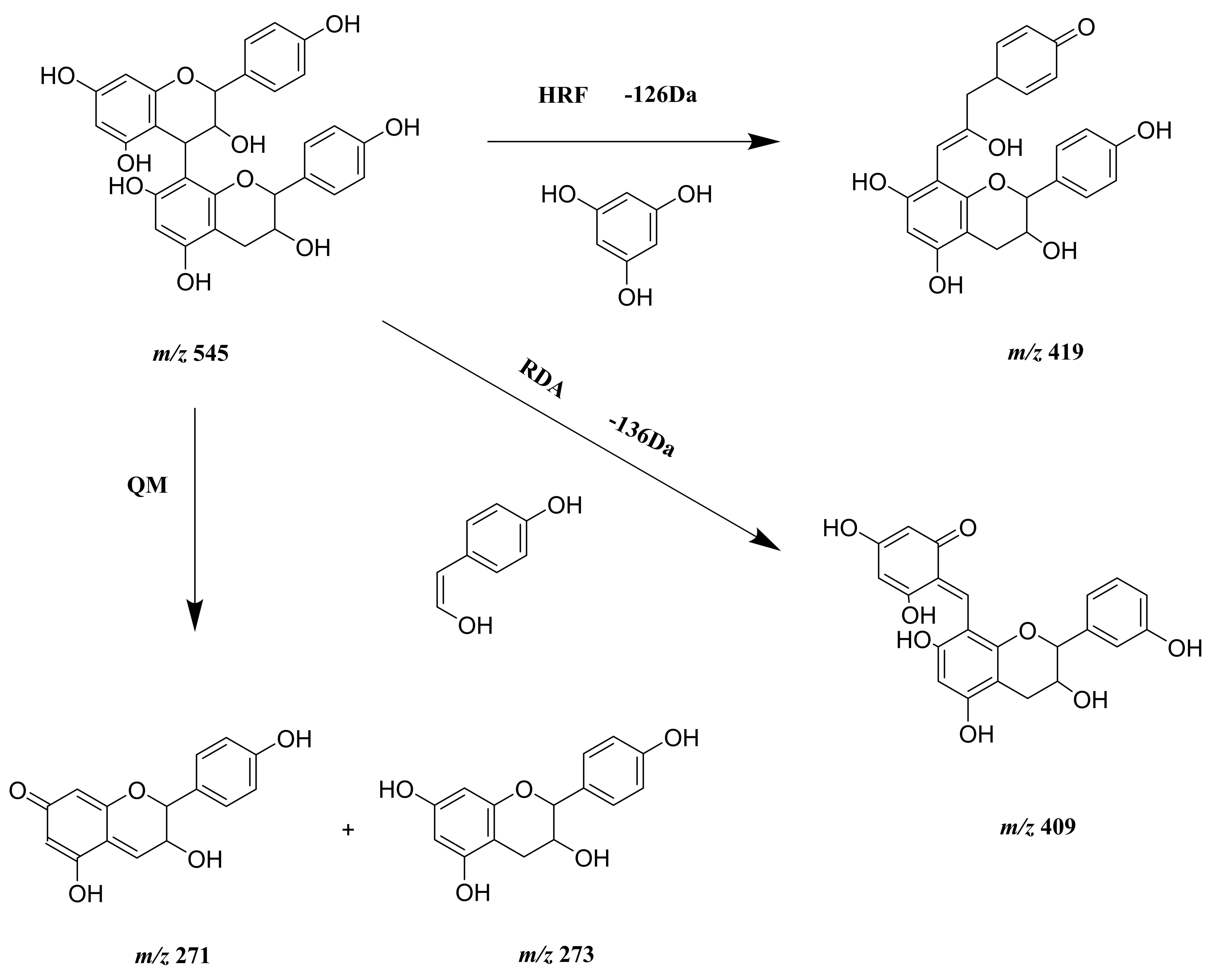


**Figure S4.** The potential fragmentation pathway of (epi)afzelechin-B-(epi)afzelechin

**Figure S5.** The potential fragmentation pathway of (epi)afzelechin-B-(epi)afzelechin-A-(epi)afzelechin


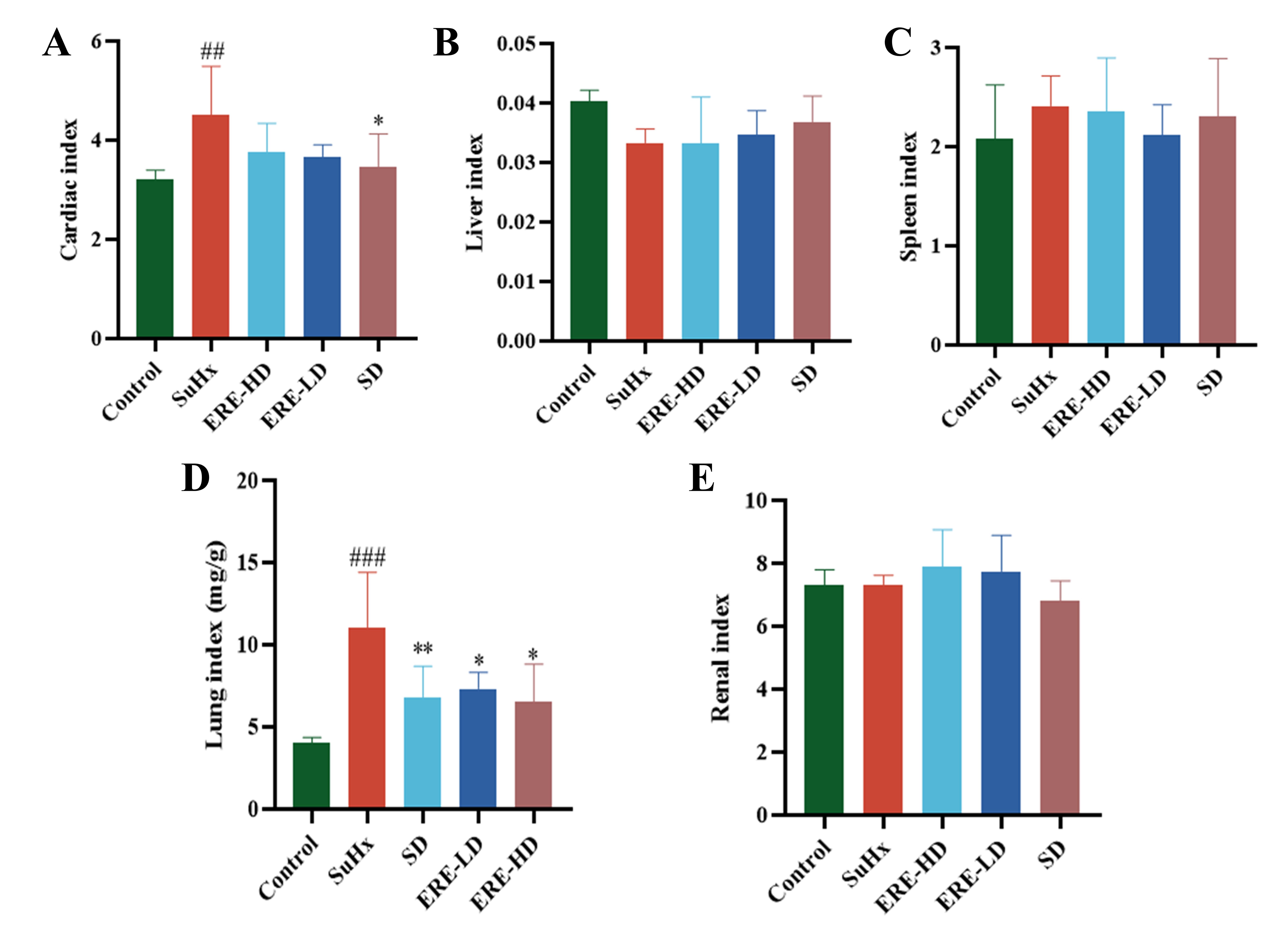


**Figure S6.** Organ index of the cardiac (A), liver (B), spleen (C), lung (D), and kidney (E) in each group of rats. ^###^p < 0.001, ^##^p < 0.01 compared with Control group; *p < 0.05, **p < 0.01, compared with SuHx group

**

**

**Figure S7.** The metabolic profile for principal component analysis (PCA) (A: ESI (+): R^2^X = 0.406, Q^2^ = 0.240 ; B: ESI (-): R^2^X = 0.428, Q^2^ = 0.267 )

**
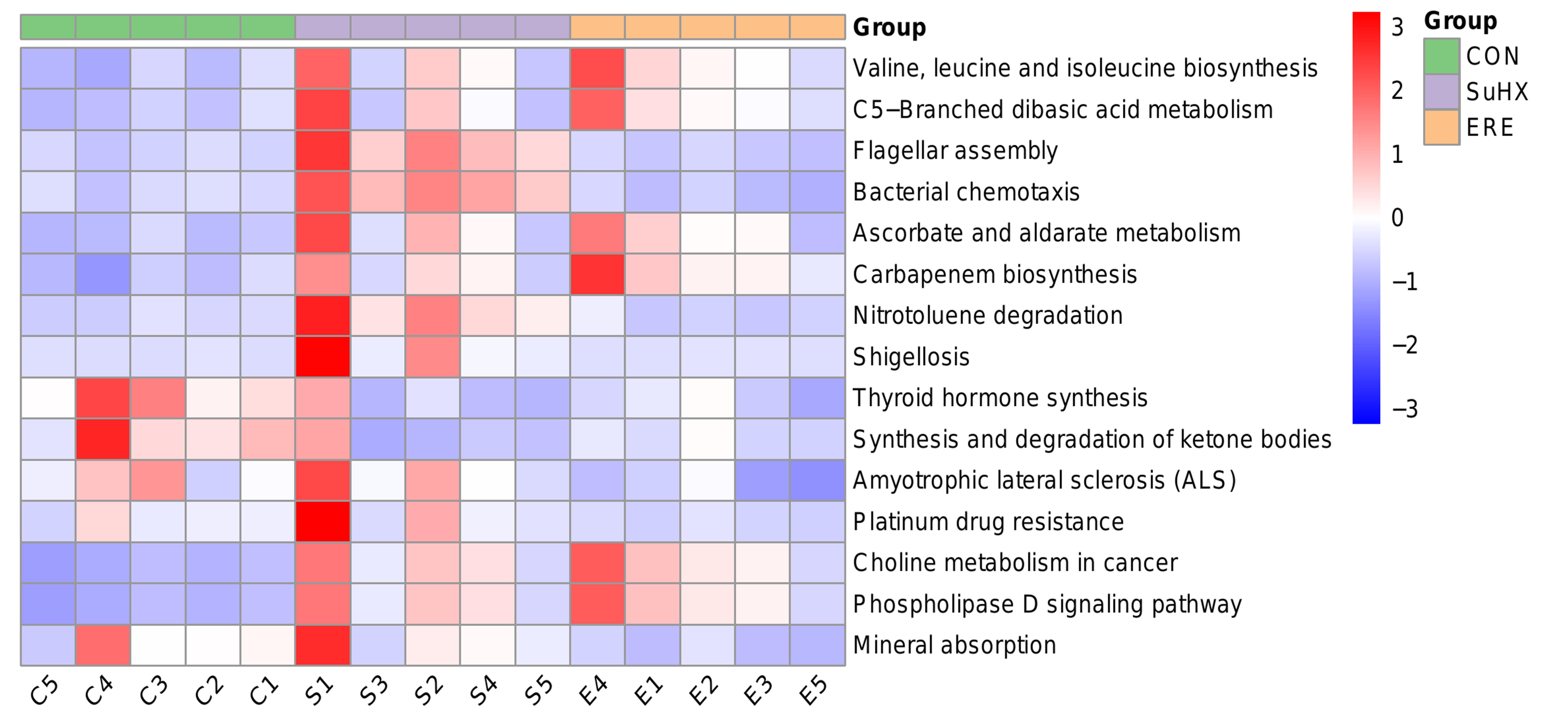
**

**Figure S8. The functional potential of the altered gut microbiota based on KEGG database**


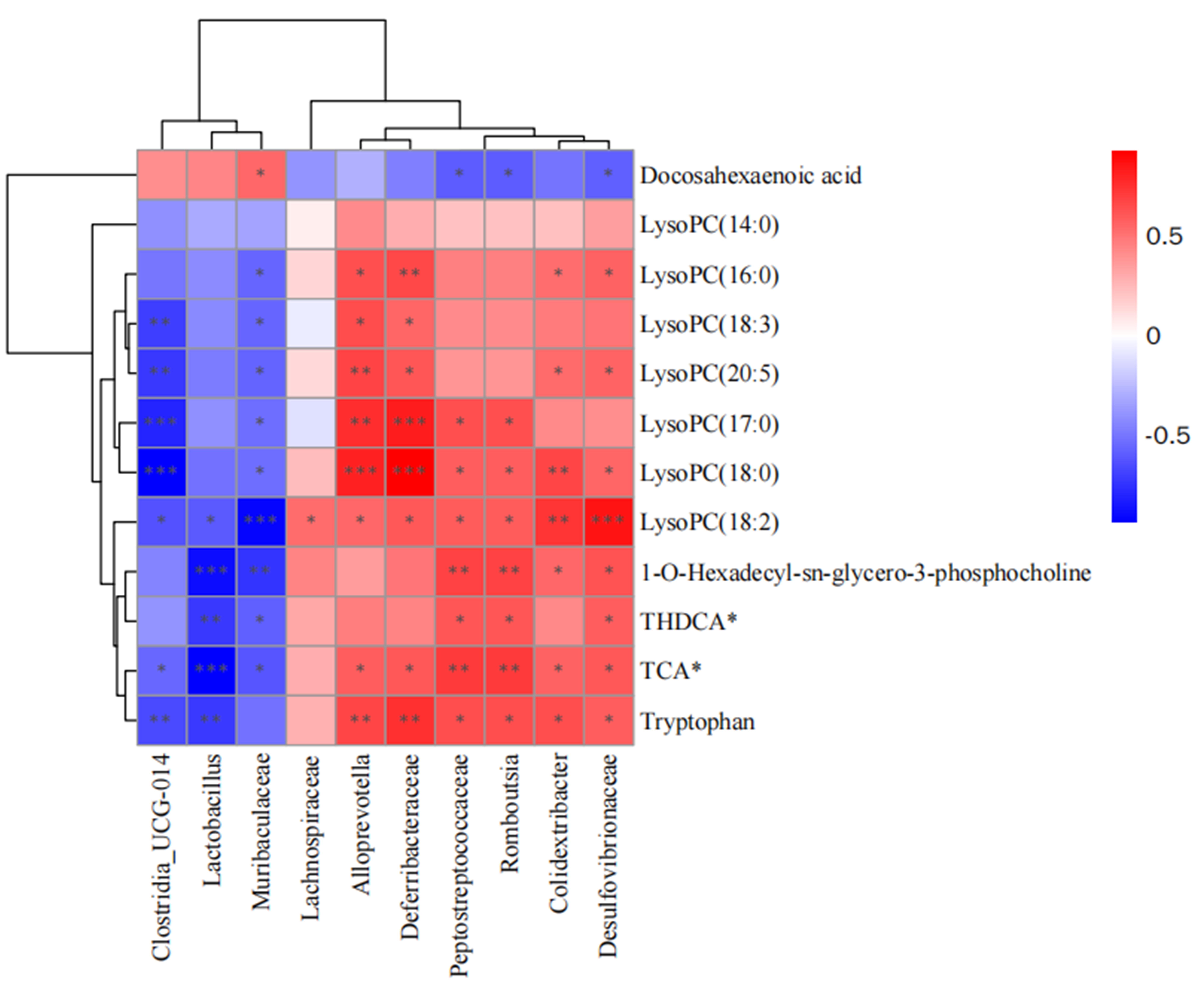


**Figure S9. The integrative correlation analysis between the significantly altered gut microbiota and the significantly changed metabolites**

**Table S1.** Tentative identification of proanthocyanidins in MH1

| **No.** | **Code** | **[M-H]^-^ *m/z*** | **T_R_(min)** | **M.F.** | **Fragment ions** | **Tentative identification** |
| --- | --- | --- | --- | --- | --- | --- |
| **1** | 273-2 | 273.0774 | 3.71 | C_15_H_14_O_5_ | 229.0872,205.0876,189.0564,187.0771,161.0616,147.0459,137.0252,123.0459,97.0301 | (epi)afzelechin |
| **2** | 273-3 | 273.0774 | 3.9 | C_15_H_14_O_5_ | 205.0876,189.0563,161.0615,147.0459,135.0459,123.0459,97.0301 | (epi)afzelechin |
| **3** | 289-1 | 289.0723 | 2.88 | C_15_H_14_O_6_ | 245.0829,221.0826,205.0513,203.0720,179.0356,177.0563,165.0201,161.0616,137.0252,125.0251,123.0459,109.0302,97.0301 | (epi)catechin |
| **4** | 289-2 | 289.0724 | 3.08 | C_15_H_14_O_6_ | 245.0828,221.0826,203.0721,187.0408,179.0358,165.0202,137.0252,125.0252,123.0459,109.0302,97.0303 | (epi)catechin |
| **5** | 305-2 | 305.0672 | 2.81 | C_15_H_14_O_7_ | 287.0567,269.0460,241.0510,219.0671,195.0307,177.0564,165.0201,161.0252,131.0252,149.0252,17.0252,125.0251,109.0301 | (epi)gallocatechin |
| **6** | 305-3 | 305.0675 | 3 | C_15_H_14_O_7_ | 287.0569,269.0464,241.0511,219.0670,195.0308,177.0565、165.0202,161.0253,149.0253,137.0253,125.0252,121.0303,109.0303 | (epi)gallocatechin |
| **7** | 527-1 | 527.1355 | 5.4 | C_30_H_24_O_9_ | 401.1037,391.0831,273.0776,253.0514 | apigeniflavan-A-(epi) afzelechin |
| **8** | 527-2 | 527.1349 | 5.6 | C_30_H_24_O_9_ | 401.1032,391.0827,273.0773,253.0511 | apigeniflavan-A-(epi)afzelechin |
| **9** | 543-1 | 543.1302 | 4.05 | C_30_H_24_O_10_ | 417.0988,407.0779,273.0776,269.0464,241.0513 | (epi)afzelechin-A-(epi)afzelechin |
| **10** | 543-2 | 543.1299 | 4.27 | C_30_H_24_O_10_ | 417.0987,273.0774,269.0463,241.0513 | (epi)afzelechin-A-(epi)afzelechin |
| **11** | 543-3 | 543.13 | 4.45 | C_30_H_24_O_10_ | 417.0989,407.0786,389.1036,273.0775,269.0464,241.0513 | (epi)afzelechin-A-(epi)afzelechin |
| **12** | 543-4 | 543.1302 | 4.72 | C_30_H_24_O_10_ | 417.0990,273.0777,269.0464,241.0514 | (epi)afzelechin-A-(epi)afzelechin |
| **13** | 543-5 | 543.1299 | 4.92 | C_30_H_24_O_10_ | 417.0988,407.0780,389.1035,273.0775,269.0464 | (epi)afzelechin-A-(epi)afzelechin |
| **14** | 543-6 | 543.1301 | 5.12 | C_30_H_24_O_10_ | 417.0989,407.0782,273.0776,269.0464 | (epi)afzelechin-A-(epi)afzelechin |
| **15** | 543-7 | 543.1299 | 5.32 | C_30_H_24_O_10_ | 417.0991,407.0782,273.0777,269.0464 | (epi)afzelechin-A-(epi)afzelechin |
| **16** | 543-8 | 543.1286 | 5.52 | C_30_H_24_O_10_ | 417.0977,407.0779,273.0775,269.0450 | (epi)afzelechin-A-(epi)afzelechin |
| **17** | 543-9 | 543.1285 | 5.72 | C_30_H_24_O_10_ | 417.0978,407.0779,273.0775,269.0445 | (epi)afzelechin-A-(epi)afzelechin |
| **18** | 545-1 | 545.1363 | 4.31 | C_30_H_26_O_10_ | 544.1331,419.1072,418.1022,273.0773,271.0521,269.0461 | (epi)afzelechin-B-(epi)afzelechin |
| **19** | 545-2 | 545.1371 | 4.6 | C_30_H_26_O_10_ | 544.1337,419.1058,418.1019,273.0776,271.0581,269.0467 | (epi)afzelechin-B-(epi)afzelechin |
| **20** | 545-3 | 545.1364 | 4.79 | C_30_H_26_O_10_ | 544.1334,418.1021,419.1022,273.0775,271.0582,269.0463 | (epi)afzelechin-B-(epi)afzelechin |
| **21** | 545-4 | 545.136 | 4.99 | C_30_H_26_O_10_ | 544.1331,419.1049,418.1017,273.0773,271.0573,269.0461 | (epi)afzelechin-B-(epi)afzelechin |
| **22** | 555-1 | 555.0933 | 6.93 | C_30_H_20_O_11_ | 469.0935,441.0989,349.0724,291.0667,285.0409,269.0463 | (epi)afzelechin-A-kaempferol |

**Table S2.** Tentative identification of proanthocyanidins in MH2

| **No.** | **Code** | **[M-H]^-^ *m/z*** | **T_R_(min)** | **M.F.** | **Fragment ions** | **Tentative identification** |
| --- | --- | --- | --- | --- | --- | --- |
| **44** | 543-1 | 543.1302 | 3.96 | C_30_H_24_O_10_ | 417.0988,407.0780,273.0775,269.0464 | (epi)afzelechin-A-(epi)afzelechin |
| **45** | 543-2 | 543.1309 | 4.16 | C_30_H_24_O_10_ | 417.0996,407.0782,273.0779,269.0467 | (epi)afzelechin-A-(epi)afzelechin |
| **10** | 543-3(MH1-10) | 543.1305 | 4.35 | C_30_H_24_O_10_ | 417.0992,407.0782,273.0778,269.0466 | (epi)afzelechin-A-(epi)afzelechin |
| **46** | 543-4 | 543.1301 | 4.58 | C_30_H_24_O_10_ | 417.0988,301.0724,273.0776,269.0464 | (epi)afzelechin-A-(epi)afzelechin |
| **12** | 543-5(MH1-12) | 543.1302 | 4.78 | C_30_H_24_O_10_ | 417.0989,273.0777,269.0464 | (epi)afzelechin-A-(epi)afzelechin |
| **13** | 543-6(MH1-13) | 543.1302 | 4.97 | C_30_H_24_O_10_ | 417.0990,407.0780,273.0776,269.0464 | (epi)afzelechin-A-(epi)afzelechin |
| **14** | 543-7(MH1-14) | 543.1303 | 5.17 | C_30_H_24_O_10_ | 417.0991,407.0783,273.0776,269.0464 | (epi)afzelechin-A-(epi)afzelechin |
| **16** | 543-8(MH1-16) | 543.1302 | 5.57 | C_30_H_24_O_10_ | 417.0987,407.0781,273.0776,269.0464 | (epi)afzelechin-A-(epi)afzelechin |
| **17** | 543-9(MH1-17) | 543.13 | 5.78 | C_30_H_24_O_10_ | 439.1196,407.0779,273.0775,269.0463 | (epi)afzelechin-A-(epi)afzelechin |
| **38** | 555(MH2-38) | 555.0925 | 10.52 | C_30_H_20_O_11_ | 417.0611,391.0829,285.0406,269.0458 | (epi)afzelechin-A-kaempferol |
| **47** | 559-1 | 559.1248 | 2.69 | C_30_H_24_O_11_ | 423.0742,389.1037,273.0775,241.0512 | (epi)catechin-A-(epi)afzelechin |
| **48** | 559-2 | 559.1234 | 2.98 | C_30_H_24_O_11_ | 433.0849,423.0726,273.0776,241.0514 | (epi)catechin-A-(epi)afzelechin |
| **49** | 559-3 | 559.1249 | 3.47 | C_30_H_24_O_11_ | 433.0939,407.0781,289.0725,269.0464 | (epi)afzelechin-A-(epi)catechin |
| **50** | 559-4 | 559.1247 | 3.67 | C_30_H_24_O_11_ | 433.0939,407.0780,289.0724,269.0463 | (epi)afzelechin-A-(epi)catechin |
| **51** | 559-5 | 559.1247 | 3.85 | C_30_H_24_O_11_ | 433.0938,407.0780,289.0724,269.0463 | (epi)afzelechin-A-(epi)catechin |
| **52** | 559-6 | 559.1251 | 4.27 | C_30_H_24_O_11_ | 433.0940,407.0782,289.0726,269.0465 | (epi)afzelechin-A-(epi)catechin |
| **53** | 559-7 | 559.1243 | 4.66 | C_30_H_24_O_11_ | 433.0938,407.0779,289.0724,269.0463 | (epi)afzelechin-A-(epi)catechin |
| **54** | 559-8 | 559.123 | 5.33 | C_30_H_24_O_11_ | 433.0933,407.0777,289.0722,269.0461 | (epi)afzelechin-A-(epi)catechin |
| **55** | 575 | 575.119 | 2.73 | C_30_H_24_O_12_ | 449.0862,289.0722,285.0400 | (epi)catechin-A-(epi)catechin |

**Table S3.** Tentative identification of proanthocyanidins in MH3

| **No.** | **Code** | **[M-H]^-^ *m/z*** | **T_R_(min)** | **M.F.** | **Fragment ions** | **Tentative identification** |
| --- | --- | --- | --- | --- | --- | --- |
| **8** | 527-1（MH1-8） | 527.1351 | 5.56 | C_30_H_24_O_10_ | 401.1035,391.1035,273.0775,253.0513 | apigeniflavan-A-(epi)afzelechin |
| **23** | 527-2 | 527.1358 | 8.5 | C_30_H_24_O_10_ | 401.1037,391.0832273.0776,253.0514 | apigeniflavan-A-(epi)afzelechin |
| **24** | 527-3 | 5.27.1353 | 8.68 | C_30_H_24_O_10_ | 401.1036,391.0832,273.0775,253.0514 | apigeniflavan-A-(epi)afzelechin |
| **25** | 529-1 | 529.1424 | 8.54 | C_30_H_26_O_10_ | 528.1384,403.1095,402.1067,393.0919,273.0774,255.0564,254.0545,253.0512 | apigeniflavan-B-(epi)afzelechin |
| **26** | 529-2 | 529.142 | 8.75 | C_30_H_26_O_10_ | 528.1385,403.1094,402.1068,393.0924,392.0865,273.0775,255.0562,254.0546,253.0513 | apigeniflavan-B-(epi)afzelechin |
| **27** | 543-1 | 543.1301 | 4.37 | C_30_H_24_O_10_ | 417.0987,407.0781,273.0776,269.0464 | apigeniflavan-A-(epi)catechin |
| **28** | 543-2 | 543.1296 | 4.6 | C_30_H_24_O_10_ | 417.1459,407.0778,273.0773,269.0461 | apigeniflavan-A-(epi)catechin |
| **12** | 543-3(MH1-12) | 543.1301 | 4.79 | C_30_H_24_O_10_ | 417.0989,407.0788,273.0776,269.0464 | (epi)afzelechin-A-(epi)afzelechin |
| **13** | 543-4(MH1-13) | 543.1301 | 5 | C_30_H_24_O_10_ | 417.0989,407.0781,273.0775,269.0464 | (epi)afzelechin-A-(epi)afzelechin |
| **17** | 543-5(MH1-17) | 543.1295 | 5.67 | C_30_H_24_O_10_ | 407.0780,273.0775,269.0461 | (epi)afzelechin-A-(epi)afzelechin |
| **29** | 543-6 | 543.1295 | 6.05 | C_30_H_24_O_10_ | 417.0984,407.0776,273.0773,269.0461 | (epi)afzelechin-A-(epi)afzelechin |
| **30** | 543-7 | 543.1301 | 7.28 | C_30_H_24_O_10_ | 417.1301,407.0780,289.0724,253.0513 | (epi)afzelechin-A-(epi)afzelechin |
| **31** | 543-8 | 543.1299 | 7.5 | C_30_H_24_O_16_ | 417.0986,407.0780,289.0724,253.0513 | (epi)afzelechin-A-(epi)afzelechin |
| **32** | 545-1 | 545.1374 | 7.38 | C_30_H_26_O_16_ | 544.1337,419.1060,418.1020,409.0840,408.0816,290.0758,289.0725,255.0561,254.0547,253.0514 | apigeniflavan-B-(epi)catechin |
| **33** | 545-2 | 545.1365 | 7.58 | C_30_H_26_O_16_ | 544.1334,419.1058,418.1018,290.0757,289.0723,255.0567,254.054,253.0513 | apigeniflavan-B-(epi)catechin |
| **34** | 555-1 | 555.0934 | 6.82 | C_30_H_20_O_11_ | 469.0937,441.0988,349.0725,285.0411,269.0464 | (epi)afzelechin-A-kaempferol |
| **35** | 555-2 | 555.0938 | 7.03 | C_30_H_20_O_11_ | 469.0941,441.0990,349.0726,285.0412,269.0464 | (epi)afzelechin-A-kaempferol |
| **36** | 555-3 | 555.0938 | 7.2 | C_30_H_20_O_11_ | 469.0936,349.0727,285.0412,269.0466 | (epi)afzelechin-A-kaempferol |
| **37** | 555-4 | 555.0939 | 10.37 | C_30_H_20_O_11_ | 469.0938,349.0727,285.0413,269.0464 | (epi)afzelechin-A-kaempferol |
| **38** | 555-5 | 555.0934 | 10.55 | C_30_H_20_O_11_ | 469.0938,349.0724,285.0410,269.0463 | (epi)afzelechin-A-kaempferol |
| **39** | 559-1 | 559.1242 | 2.76 | C_30_H_24_O_11_ | 515.134,423.0728,285.0405,273.0772,241.0510 | (epi)catechin-A-(epi)afzelechin |
| **40** | 559-2 | 559.1245 | 2.94 | C_30_H_24_O_11_ | 515.1351,423.0730,285.0407,273.0773,241.0510 | (epi)catechin-A-(epi)afzelechin |
| **41** | 559-3 | 559.1247 | 3.01 | C_30_H_24_O_11_ | 515.1325,423.0730,389.1035,273.0774 | (epi)catechin-A-(epi)afzelechin |
| **42** | 559-4 | 559.1232 | 4.68 | C_30_H_24_O_11_ | 433.0933,407.0770,289.0721,269.0460 | (epi)afzelechin-A-(epi)catechin |
| **43** | 559-5 | 559.1243 | 4.83 | C_30_H_24_O_11_ | 559.1230,433.0933,407.0774,289.0721,269.0461 | (epi)afzelechin-A-(epi)catechin |

**Table S4.** Tentative identification of proanthocyanidins in MH4

| **No.** | **Code** | **[M-H]^-^ *m/z*** | **T_R_(min)** | **M.F.** | **Fragment ions** | **Tentative identification** |
| --- | --- | --- | --- | --- | --- | --- |
| **44** | 543-1 | 543.1302 | 3.96 | C_30_H_24_O_10_ | 417.0988,407.0780,273.0775,269.0464 | (epi)afzelechin-A-(epi)afzelechin |
| **45** | 543-2 | 543.1309 | 4.16 | C_30_H_24_O_10_ | 417.0996,407.0782,273.0779,269.0467 | (epi)afzelechin-A-(epi)afzelechin |
| **10** | 543-3(MH1-10) | 543.1305 | 4.35 | C_30_H_24_O_10_ | 417.0992,407.0782,273.0778,269.0466 | (epi)afzelechin-A-(epi)afzelechin |
| **46** | 543-4 | 543.1301 | 4.58 | C_30_H_24_O_10_ | 417.0988,301.0724,273.0776,269.0464 | (epi)afzelechin-A-(epi)afzelechin |
| **12** | 543-5(MH1-12) | 543.1302 | 4.78 | C_30_H_24_O_10_ | 417.0989,273.0777,269.0464 | (epi)afzelechin-A-(epi)afzelechin |
| **13** | 543-6(MH1-13) | 543.1302 | 4.97 | C_30_H_24_O_10_ | 417.0990,407.0780,273.0776,269.0464 | (epi)afzelechin-A-(epi)afzelechin |
| **14** | 543-7(MH1-14) | 543.1303 | 5.17 | C_30_H_24_O_10_ | 417.0991,407.0783,273.0776,269.0464 | (epi)afzelechin-A-(epi)afzelechin |
| **16** | 543-8(MH1-16) | 543.1302 | 5.57 | C_30_H_24_O_10_ | 417.0987,407.0781,273.0776,269.0464 | (epi)afzelechin-A-(epi)afzelechin |
| **17** | 543-9(MH1-17) | 543.13 | 5.78 | C_30_H_24_O_10_ | 439.1196,407.0779,273.0775,269.0463 | (epi)afzelechin-A-(epi)afzelechin |
| **38** | 555(MH2-38) | 555.0925 | 10.52 | C_30_H_20_O_11_ | 417.0611,391.0829,285.0406,269.0458 | (epi)afzelechin-A-kaempferol |
| **47** | 559-1 | 559.1248 | 2.69 | C_30_H_24_O_11_ | 423.0742,389.1037,273.0775,241.0512 | (epi)catechin-A-(epi)afzelechin |
| **48** | 559-2 | 559.1234 | 2.98 | C_30_H_24_O_11_ | 433.0849,423.0726,273.0776,241.0514 | (epi)catechin-A-(epi)afzelechin |
| **49** | 559-3 | 559.1249 | 3.47 | C_30_H_24_O_11_ | 433.0939,407.0781,289.0725,269.0464 | (epi)afzelechin-A-(epi)catechin |

**Table S5.** Tentative identification of proanthocyanidins in MH5

| **No.** | **Code** | **T_R_(min)** | **[M-H]^-^ *m/z*** | **M.F.** | **Fragment ions** | **Tentative identification** |
| --- | --- | --- | --- | --- | --- | --- |
| **10** | 543-1(MH1-10) | 4.32 | 543.1297 | C_30_H_24_O_10_ | 417.0984,273.0772,269.0461,125.0251 | (epi)afzelechin-A-(epi)afzelechin |
| **64** | 543-2 | 5.66 | 543.1295 | C_30_H_24_ O_10_ | 407.0776,273.0774,269.0459,125.0251 | (epi)afzelechin-A-(epi)afzelechin |
| **65** | 575-1 | 3.02 | 575.1194 | C_30_H_24_O_12_ | 449.0885,423.0727,407.0779,89.0723,285.0410,125.0252 | (epi) catechin-A-(epi)catechin |
| **66** | 575-2 | 3.34 | 575.1194 | C_30_H_24_O_12_ | 449.0887,423.0728,407.0780,289.0723,285.0411,125.0252 | (epi) catechin-A-(epi)catechin |
| **67** | 575-3 | 3.61 | 575.1193 | C_30_H_24_O_12_ | 449.0888,407.0779,305.0672,289.0724,285.0411,269.0463,125.0252 | (epi) catechin-A-(epi)catechin |
| **68** | 575-4 | 3.83 | 575.1197 | C_30_H_24_O_12_ | 449.0886,423.0727,407.0778,391.0829,289.0723,285.0411,125.0252 | (epi) catechin-A-(epi)catechin |
| **69** | 577-1 | 2.33 | 577.1344 | C_30_H_26_O_12_ | 451.1038,425.0878,407.0774,289.0718,287.0564 | (epi) catechin-B-(epi)catechin |
| **70** | 577-2 | 2.53 | 577.1352 | C_30_H_26_O_12_ | 451.1038,425.0878,407.0771,289.0719,287.0562 | (epi) catechin-B-(epi)catechin |
| **71** | 577-3 | 2.59 | 577.1346 | C_30_H_26_O_12_ | 451.1046,425.0881,407.0772,289.0720,287.0563 | (epi) catechin-B-(epi)catechin |
| **72** | 577-4 | 2.61 | 577.1348 | C_30_H_26_O_12_ | 451.1046,425.0881,407.0772,289.0720,287.0563 | (epi) catechin-B-(epi)catechin |
| **73** | 591-1 | 2.65 | 591.1137 | C_30_H_24_O_13_ | 555.0938,465.0839,439.0679,301.0360,289.0723,191.0357,175.0044,125.0252 | (epi)gallocatechin-A-(epi)catechin |
| **74** | 591-2 | 2.85 | 591.1134 | C_30_H_24_O_13_ | 555.0939,465.0836,439.0680,301.0360,289.0723,285.0411,125.0252 | (epi)gallocatechin-A-(epi)catechin |
| **75** | 591-3 | 3.068 | 591.1142 | C_30_H_24_O_13_ | 555.0938,465.0837,439.0676,301.0360,289.0723,191.0357,175.0044,125.0252 | (epi)gallocatechin-A-(epi)catechin |
| **76** | 591-4 | 3.464 | 591.1134 | C_30_H_24_O_13_ | 555.0937,465.0836,439.0681,301.0359,289.0723,191.0356,175.0044,125.0251 | (epi)gallocatechin-A-(epi)catechin |
| **77** | 813-1 | 4.21 | 813.1812 | C_45_H_36_O_15_ | 677.1301,543.1302,417.1464,273.0774,269.0461 | (epi)afzelechin-A-(epi)afzelechin-A-(epi)afzelechin |
| **78** | 813-2 | 5.08 | 813.1817 | C_45_H_36_O_15_ | 677.1302,543.1302,273.0775,269.0463 | (epi)afzelechin-A-(epi) afzelechin-A-(epi) afzelechin |
| **79** | 813-3 | 5.29 | 813.1807 | C_45_H_36_O_15_ | 677.1302,543.1296,417.0987,273.0772,269.0458 | (epi)afzelechin-A-(epi)afzelechin-A-(epi)afzelechin |
| **80** | 813-4 | 5.49 | 813.1887 | C_45_H_36_O_15_ | 677.1307,543.1296,417.0984,273.0772,269.0459 | (epi)afzelechin-A-(epi)afzelechin-A-(epi)afzelechin |
| **81** | 815-1 | 3.56 | 815.1976 | C_45_H_38_O_15_ | 679.1470、543.1309、541.1149、434.0654、407.0773、312.0648、273.0775、269.0464 | (epi)afzelechin-B-(epi) afzelechin-A-(epi) afzelechin |
| **82** | 815-2 | 3.77 | 815.1962 | C_45_H_38_O_15_ | 689.1653、679.1463、543.1302、407.0778、312.0647、273.0775、269.0463、125.0252 | (epi)afzelechin-B-(epi) afzelechin-A-(epi) afzelechin |
| **83** | 815-3 | 5.37 | 815.1962 | C_45_H_38_O_15_ | 689.1673、679.1459、543.1299、417.0985、312.0647、301.0722、273.0773、269.0461、125.0251 | (epi)afzelechin-B-(epi) afzelechin-A-(epi) afzelechin |
| **84** | 815-4 | 5.61 | 815.1956 | C_45_H_38_O_15_ | 689.1676、679.1443、543.1299、417.0985、407.0762、273.0773、269.0462 | (epi)afzelechin-B-(epi) afzelechin-A-(epi) afzelechin |
| **85** | 817-1 | 3.74 | 817.2033 | C_45_H_40_O_15_ | 816.2006、680.1502、545.1364、544.1337、407.0778273.0776、271.0627、269.0463、 | (epi)afzelechin-B-(epi) afzelechin-B-(epi) afzelechin |
| **86** | 817-2 | 5.46 | 817.2032 | C_45_H_40_O_15_ | 816.1998、690.1702、545.1362、544.1335、273.0775、271.0624、269.0462 | (epi)afzelechin-B-(epi) afzelechin-B-(epi) afzelechin |
| **87** | 817-3 | 5.64 | 817.2034 | C_45_H_40_O_15_ | 816.1999、690.1711、545.1365、544.1336、273.0775、271.0623、269.0463 | (epi)afzelechin-B-(epi) afzelechin-B-(epi) afzelechin |

**Table S6.** Parameters of the OPLS-DA model

| OPLS-DA | Serum Sample ESI (+) | | Serum Sample ESI (-) | |
| --- | --- | --- | --- | --- |
|  | Control | SuHx | Control | SuHx |
|  | vs.  SuHx | vs.  ERE-HD | vs.  SuHx | vs.  ERE-HD |
| A | 1+2+0 | 1+1+0 | 1+2+0 | 1+2+0 |
| R^2^X(cum) | 0.564 | 0.581 | 0.560 | 0.543 |
| R^2^Y(cum) | 0.993 | 0.963 | 0.995 | 0.990 |
| Q^2^(cum) | 0.909 | 0.855 | 0.848 | 0.757 |

**Table S7.** Information on MS^2^ of potential serum biomarker

| **Metabolite** | **Formula** | **Retention** | **Measured** | **ESI** | **MS^2^** |
| --- | --- | --- | --- | --- | --- |
|  |  | **time** |  | **ionization** |  |
|  |  | (min) | **(m/z)** | **mode** |  |
| Tryptophan | C_11_H_12_N_2_O_2_ | 2.043 | 203.08192 | - | 186.0562, 159.0930, 142.0664, 116.0508, 74.0250 |
| TCA* | C_26_H_45_NO_7_S | 4.874 | 514.28192 | - | — |
| THDCA* | C_26_H_45_NO_6_S | 5.269 | 498.28714 | - | 372.2957 |
| LysoPC(14:0) | C_22_H_46_NO_7_P | 7.618 | 468.30751 | + | 450.2977, 285.2426, 184.0734, 104.1068, 86.0962 |
| LysoPC(20:5) | C_28_H_48_NO_7_P | 7.679 | 542.32299 | + | 524.3134, 258.1101, 240.0993, 184.0732, 124.9997, 104.1068, 86.0962 |
| LysoPC(18:3) | C_26_H_48_NO_7_P | 7.742 | 518.3232 | + | 500.3130, 258.1100, 240.0996, 184.0732, 124.9997, 104.1068, 86.0962 |
| LysoPC(18:2) | C_26_H_50_NO_7_P | 8.212 | 520.33659 | + | 502.3295, 258.1104, 240.1000, 184.0734, 124.9997, 104.1068, 86.0962 |
| LysoPC(16:0) | C_24_H_50_NO_7_P | 8.593 | 496.33655 | + | 478.3292, 258.1104, 240.0996, 184.0733, 124.9997, 104.1068, 86.0962 |
| 1-O-Hexadecyl-sn-glycero-3-phosphocholine | C_24_H_52_NO_6_P | 8.827 | 482.3592 | + | 299.2945, 184.0734, 84.0807 |
| LysoPC(18:0) | C_26_H_54_NO_6_P | 9.036 | 508.37512 | + | 490.3652, 240.0999, 184.0734, 104.1068, 86.0962 |
| LysoPC(17:0) | C_25_H_52_NO_7_P | 9.054 | 510.35421 | + | 492.3447, 184.0733, 104.1068, 86.0962 |
| Docosahexaenoic acid | C_22_H_32_O_2_ | 10.378 | 329.24707 | + | 311.2370, 301.2162, 269.2264, 247.1692 |

*: Metabolites identified by reference standards; others were identified by exact molecular mass or MS^2^
